# Abnormal platelet parameters in inflammatory bowel disease: a systematic review and meta-analysis

**DOI:** 10.1186/s12876-024-03305-9

**Published:** 2024-07-03

**Authors:** Cheng Xu, Zhen Song, Li-ting Hu, Yi-heng Tong, Jing-yi Hu, Hong Shen

**Affiliations:** 1 Affiliated Hospital of Nanjing University of Chinese Medicine, Nanjing, China; 2 Nanjing University of Chinese Medicine, Nanjing, China; 3 Yancheng Binhai Hospital of Traditional Chinese Medicine, Yancheng, China

**Keywords:** Inflammatory bowel disease, Platelet parameters, Platelet count, Mean platelet volume, Platelet distribution width, Plateletcrit, Systematic review, Meta-analysis

## Abstract

**Background:**

Platelet dysfunction plays a critical role in the pathogenesis of inflammatory bowel disease (IBD). Despite clinical observations indicating abnormalities in platelet parameters among IBD patients, inconsistencies persist, and these parameters lack standardization for diagnosis or clinical assessment.

**Methods:**

A comprehensive search was conducted in the PubMed, Embase, Web of Science, and Cochrane Library databases for relevant articles published up to December 16th, 2023. A random-effects model was employed to pool the weighted mean difference (WMD) and 95% confidence interval (95% CI) of platelet count (PLT), mean platelet volume (MPV), platelet distribution width (PDW), and plateletcrit (PCT) between IBD patients and healthy controls, and subgroup analyses were performed.

**Results:**

The meta-analysis included 79 articles with 8,350 IBD patients and 13,181 healthy individuals. The results revealed significantly increased PLT and PCT levels (WMD: 69.910, 95% CI: 62.177, 77.643 10^9^/L; WMD: 0.046%, 95% CI: 0.031%, 0.061%), and decreased MPV levels (WMD: -0.912, 95% CI: -1.086, -0.739 fL) in IBD patients compared to healthy individuals. No significant difference was found in PDW between the IBD and control groups (WMD: -0.207%, 95% CI: -0.655%, 0.241%). Subgroup analysis by disease type and disease activity showed no change in the differences for PLT, PCT, and MPV in the ulcerative colitis and Crohn’s disease groups, as well as the active and inactive groups. Notably, the active group exhibited significantly lower PDW levels than the control group (WMD: -1.138%, 95% CI: -1.535%, -0.741%).

**Conclusions:**

Compared with healthy individuals, IBD patients display significantly higher PLT and PCT and significantly lower MPV. Monitoring the clinical manifestations of platelet abnormalities serves as a valuable means to obtain diagnostic and prognostic information. Conversely, proactive measures should be taken to prevent the consequences of platelet abnormalities in individuals with IBD.

**Systematic review registration:**

PROSPERO CRD42023493848.

**Supplementary Information:**

The online version contains supplementary material available at 10.1186/s12876-024-03305-9.

## Key messages

• It is known that inflammatory bowel disease (IBD) is related to various alterations in platelets, but there are variations in reported results of platelet parameters in the IBD population in clinical studies.

• To elucidate the diagnostic and prognostic value of platelet parameters in IBD, we conducted a meta-analysis of 79 studies to provide more comprehensive conclusions on changes in platelet parameters in IBD.

• This meta-analysis reveals that individuals with IBD exhibit significantly elevated platelet count (PLT) and plateletcrit (PCT), alongside a significant decrease in mean platelet volume (MPV) compared to healthy individuals.

• The platelet parameters can offer reliable insights for assessing the severity of IBD and understanding potential pathophysiological mechanisms.

**Supplementary Information:**

The online version contains supplementary material available at 10.1186/s12876-024-03305-9.

## Introduction

Inflammatory bowel disease (IBD) is a chronic immune-mediated inflammation of the intestinal tract, characterized by recurring periods of relapse and remission [1]. It encompasses two primary disorders, ulcerative colitis (UC) and Crohn’s disease (CD), both of which have demonstrated a global increase in incidence over the past decade [2–4]. Although the etiology remains incompletely elucidated, Growing experimental and clinical evidence suggests that the initiation and progression of IBD involve an intricate interplay among immune, genetic, and environmental factors [5].

Platelets, as multifunctional cells, play a crucial role in regulating immunity and inflammation, in addition to their hemostatic function [6]. IBD is linked to various alterations in platelets, encompassing changes in number, shape, and function. These abnormalities are primarily attributed to the highly activated state of circulating platelets in IBD patients [7]. IBD patients have an imbalance between inflammation and coagulation function; inflammatory cytokines can induce abnormal coagulation processes, and the coagulation cascade promotes the progression of inflammation, initiating a vicious cycle where each process propagates and intensifies the other [8, 9]. The significance of activated platelets in the pathogenesis of IBD becomes more pronounced when considering that patients with IBD are prone to developing arterial thromboembolic events and venous thromboembolism [10, 11].

While platelet dysfunction is implicated in IBD pathogenesis, the existing evidence remains insufficient. Moreover, there are variations in reported results of platelet parameters in the IBD population in clinical studies, and platelet parameters have not yet been established as guidelines for the diagnosis or clinical evaluation of IBD. Therefore, accumulating evidence is crucial to elucidate the diagnostic and prognostic value of platelet parameters in IBD. To address this, we conducted a meta-analysis to provide more comprehensive conclusions on changes in platelet parameters in IBD, including platelet count (PLT), mean platelet volume (MPV), platelet distribution width (PDW), and plateletcrit (PCT). As a cost-effective and straightforward laboratory indicator, examining the correlation between platelet parameters and IBD has the potential to contribute to more comprehensive clinical insights for the prevention, diagnosis, and treatment of IBD and related platelet disorders.

## Methods

### Search strategy and selection criteria

This systematic review and meta-analysis was conducted in accordance with the Preferred Reporting Items for a Systematic Review and Meta-analysis (PRISMA) guideline and the Meta-analysis of Observational Studies in Epidemiology (MOOSE) reporting guideline. (Table S1, S2) [12]. The protocol for this meta-analysis was registered with PROSPERO (CRD42023493848).

We performed a thorough search in the PubMed, Embase, Web of Science, and Cochrane Library databases to identify relevant articles. Our search strategy encompassed MeSH (Medical Subject Headings) terms and entry terms without language restrictions. Furthermore, the reference lists of eligible articles were carefully screened to identify any additional relevant studies that were not initially retrieved during the literature search. The details of the search strategy are presented in Table S3.

The inclusion criteria were as follows: (1) Patients: adult IBD patients (the majority of participants > 18 years of age); (2) Control: adults in good health; (3) Outcomes: PLT, MPV, PDW, and PCT; (4) Study type: cross-sectional studies, case control studies, and cohort studies.

The exclusion criteria were as follows: (1) Animal experiments, reviews, conference abstracts, case reports and meta-analyses; (2) Research object limited to children or adolescents [3], Pregnant or lactating women, hematological diseases, malignant tumors, and any other diseases that could interfere with the platelet parameters; (3) Participants received anticoagulant medications, contraceptives, or heparin, and any other drugs unrelated to IBD treatment that may cause platelet abnormalities; (4) Study of drug efficacy (5) Full-text or sufficient data could not be extracted.

### Data collection and extraction

EndNote 20 software was utilized to manage studies to remove duplicate articles obtained from the database. The screening of included articles, based on predefined inclusion and exclusion criteria, was performed independently by CX and ZS. Resolution of any discrepancies arising from this screening process involved discussion with another author (HS) until a consensus was reached. Extracted research information encompassed: (1) Background details including first author, publication year, country, study design, sample size, age, and sex; (2) Platelet parameters including PLT, MPV, PDW, and PCT, presented as mean ± standard deviation (SD). In instances where relevant data were missing, attempts were made to contact the corresponding author to obtain the required information. If multiple groups of data were presented within the same study, each group was treated as an independent entity for data extraction.

### Quality assessment

The Newcastle-Ottawa Scale (NOS) was employed to assess the quality of cohort studies and case control studies [13], and the Agency for Healthcare Research and Quality (AHRQ) methodology checklist was used to evaluate cross-sectional studies [14]. The NOS evaluates research quality based on three aspects: selection of study groups, comparability of groups, and ascertainment of exposure or outcome of interest for case control or cohort studies, respectively **(Methods 1, 2**). Study quality was categorized as follows: low quality = 0–3 stars, moderate quality = 4–6 stars, and high quality = 7–9 stars [15]. On the other hand, the AHRQ methodology checklist comprises 11 items, including a definition of information source, inclusion and exclusion criteria, time period and continuity for identifying patients, personnel blinding, quality assurance assessments, confounding and missing data, and patient response rates and completeness (**Methods 3**). For each item, a score of “0” was assigned if the answer was “unclear” or “no”, and a score of “1” was given if the answer was “yes”. Study quality was categorized as follows: low quality = 0–3, moderate quality = 4–7, and high quality = 8–11 [16]. Two investigators (CX and SZ) independently assessed the quality of eligible articles, with any discrepancies resolved by a third investigator (HS).

### Data synthesis and data analysis

The analysis encompassed the calculation and estimation of the Weighted Mean Difference (WMD) along with its 95% Confidence Interval (95% CI) for key platelet parameters (PLT, MPV, PDW, and PCT) in IBD patients compared to healthy controls across each study. For studies utilizing median, range, and/or interquartile range, we employed the methodologies proposed by Wan et al. [17] and Luo et al. [18] to estimate the sample mean and SD. A positive WMD indicated higher platelet parameter values in IBD patients. In cases where outcome changes were not explicitly reported, conversion formulas recommended in the Cochrane Handbook Version 6.4 were applied to calculate these changes [19].

The I² statistic quantitatively assessed the heterogeneity of studies, categorized as low (0–25%), moderate (25–75%), and high (> 75%) heterogeneity. A random-effects model was applied due to high heterogeneity. Meta-regression analyses were conducted to identify potential sources of heterogeneity, considering factors such as disease type, disease activity, region, study year, study type, and quality assessment. Subgroup analyses were performed based on disease type (UC, CD, and Unclassified), disease activity (active, inactive, and Unclassified), region (Asia, Europe, North America, Africa, and Oceania), study year (Before 2000 and Since 2000) study type (Case control study, Cohort study, and Cross-sectional study), and quality assesment (High quality and Moderate quality). Through this comprehensive exploration, we aim to clarify whether variations in disease characteristics, geographic location, temporal trends, study design, and study quality contribute to the heterogeneity among studies.

Moreover, sensitivity analysis was conducted, systematically removing one study at a time to assess the robustness of the results. Funnel plots and Egger’s test were employed to scrutinize publication bias. In instances of detected publication bias, the trim and fill method was applied to rectify funnel plot asymmetry.

All statistical analyses were conducted using Stata Version 12.0, and significance was defined as a 2-tailed *P* value < 0.05.

## Results

### Search and selection result

**Fig. 1:**
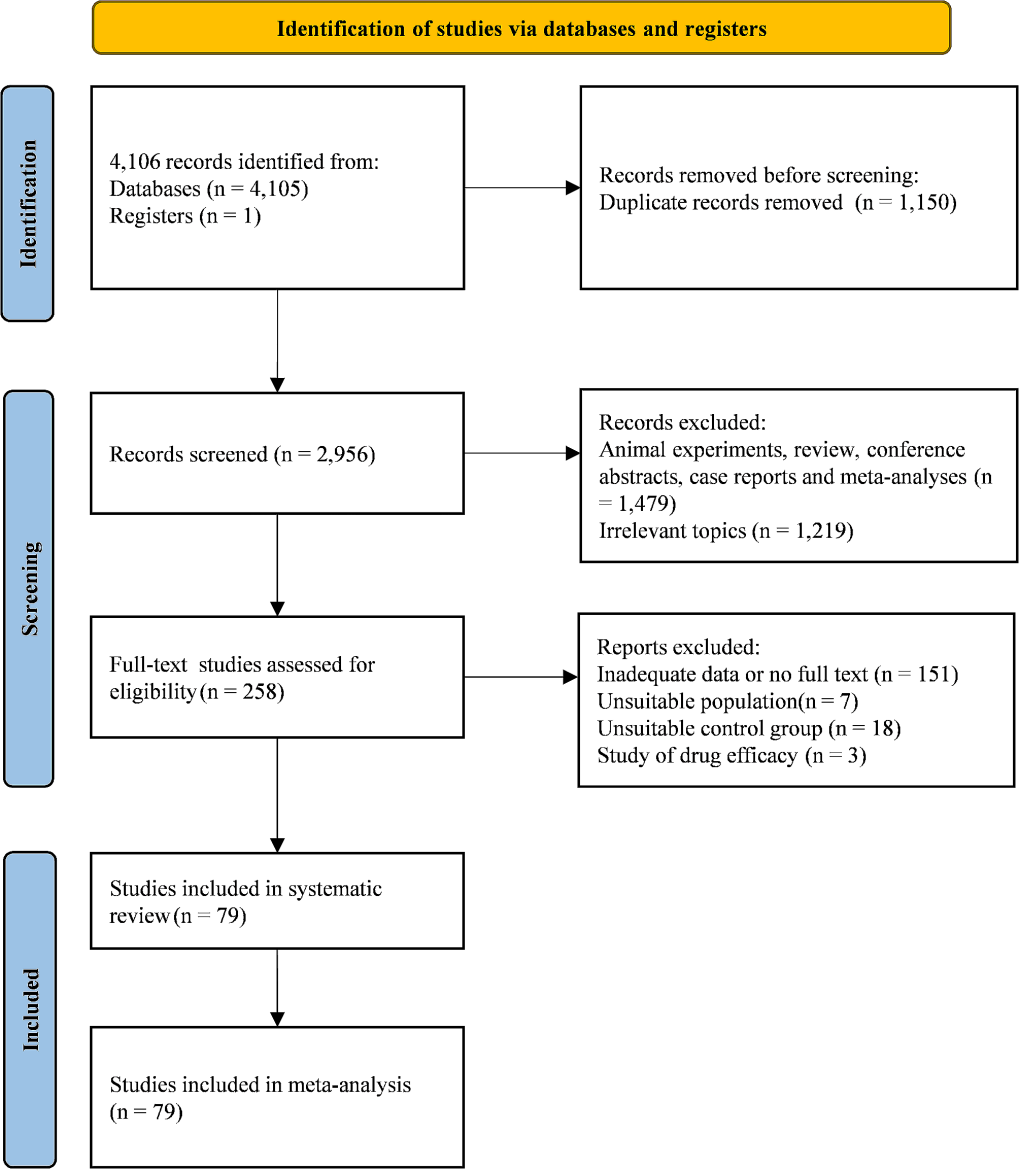
Flow diagram of study selection

In our initial search, a total of 4,106 publications were identified, from which 1,150 duplicate records were eliminated. Following the screening of the remaining 2,956 records, we excluded studies involving animal experiments, reviews, meeting abstracts, case reports, or meta-analyses, resulting in a total exclusion of 1,479 publications. Additionally, 1,219 articles were deemed irrelevant based on title and abstract assessment. Subsequently, 258 publications remained for a thorough evaluation of the full text. Of those 258 studies, 151 were excluded because of inadequate data or no full text, 8 articles were found to be unsuitable populations, 18 articles were removed because of their unsuitable control group, and 3 articles were disregarded due to their focus on drug efficacy. Finally, 79 eligible articles have been retained for inclusion in this systematic review and meta-analysis [20–98]. The specific screening process is shown in Fig. 1.

### Studies characteristics and quality assessment

The characteristics of 79 studies included in this systematic review and meta-analysis are illustrated in Table S4. These studies, spanning the period from 1975 to 2023, were conducted globally. One study adopted a gender-stratified approach [45], while the remaining studies encompassed both genders. Among the 79 studies, there were 20 case control studies, 8 cohort studies, and 51 cross-sectional studies. Based on the NOS and AHRQ methodology checklist, 70 articles were categorized as high quality, with the remaining 9 articles considered of moderate quality. Detailed assessment scores are shown in Table S4.

### Meta-analyses

**Fig. 2:**
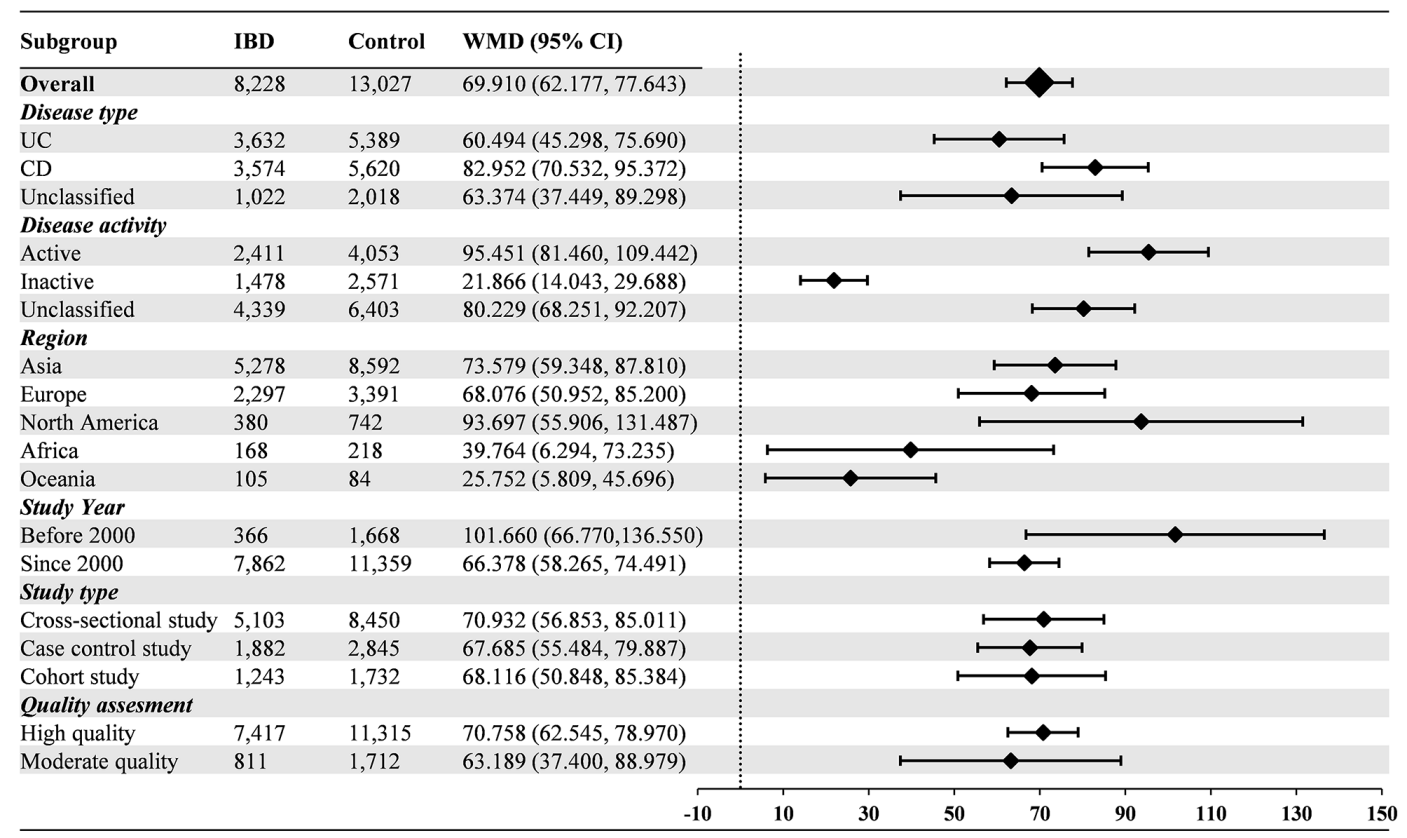
Weighted Mean Difference in PLT between the IBD Group and the Control Group. PLT measurements are reported in 10^9^/L. Abbreviations: IBD: inflammatory bowel disease; UC: ulcerative colitis; CD: Crohn’s disease; PLT: platelet count; WMD: weighted mean difference; 95% CI: 95% confidence interval

**Fig. 3:**
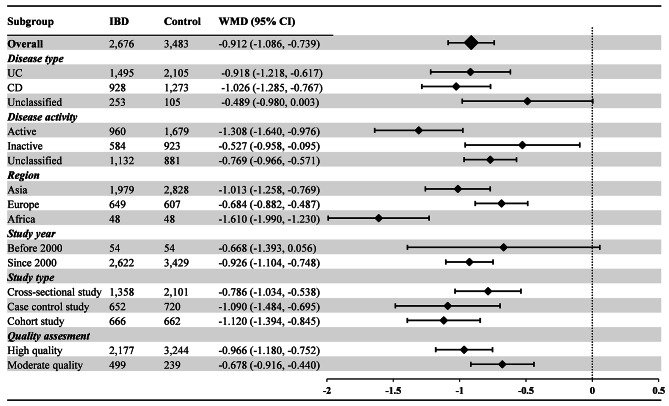
Weighted Mean Difference in MPV between the IBD Group and the Control Group. MPV measurements are reported in femtoliters (fL). Abbreviations: IBD: inflammatory bowel disease; UC: ulcerative colitis; CD: Crohn’s disease; MPV: mean platelet volume; WMD: weighted mean difference; 95% CI: 95% confidence interval

**Fig. 4:**
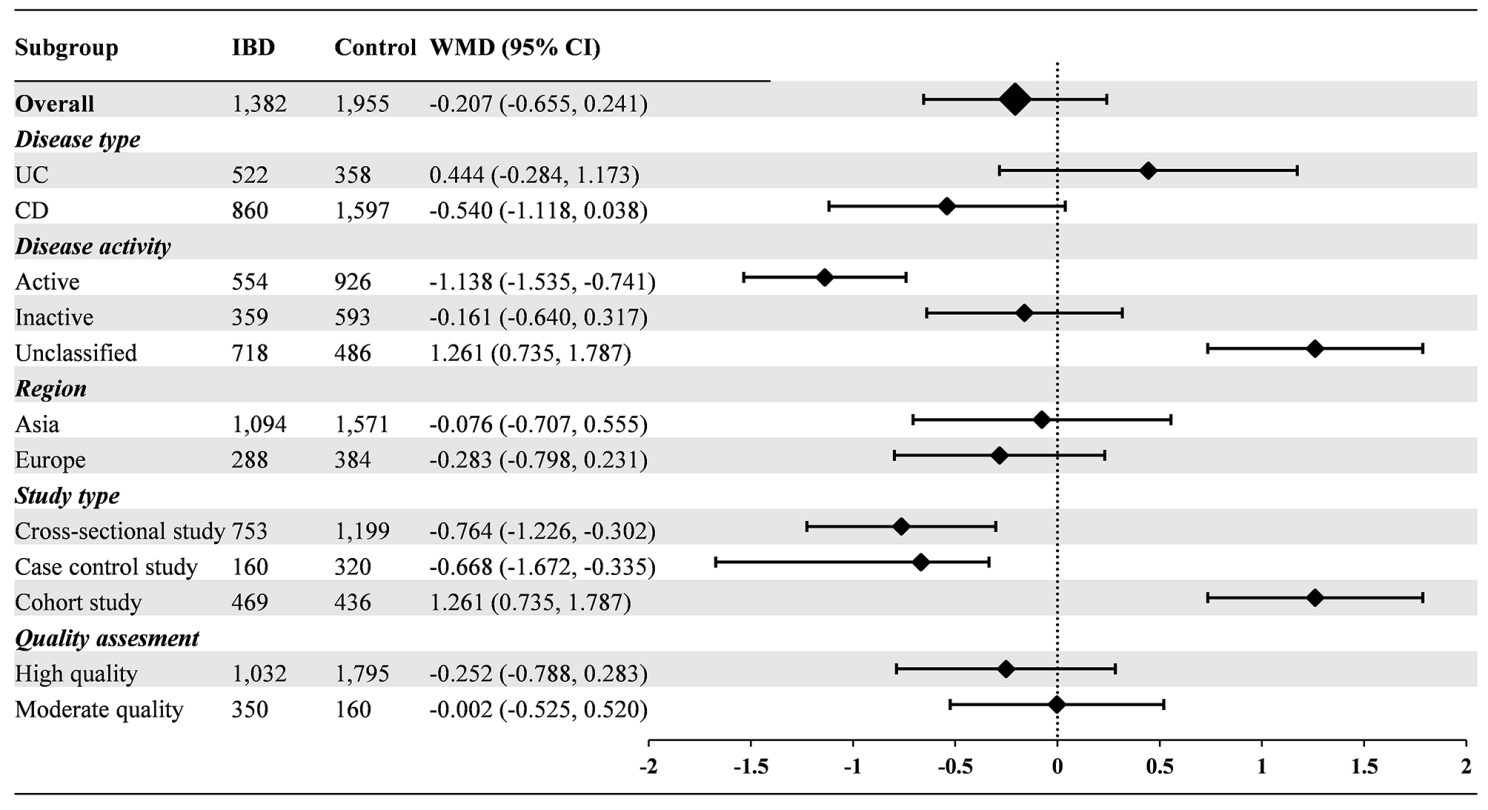
Weighted Mean Difference in PDW between the IBD Group and the Control Group. PDW measurements are reported in percentage (%). Abbreviations: IBD: inflammatory bowel disease; UC: ulcerative colitis; CD: Crohn’s disease; PDW: platelet distribution width; WMD: weighted mean difference; 95% CI: 95% confidence interval

**Fig. 5:**
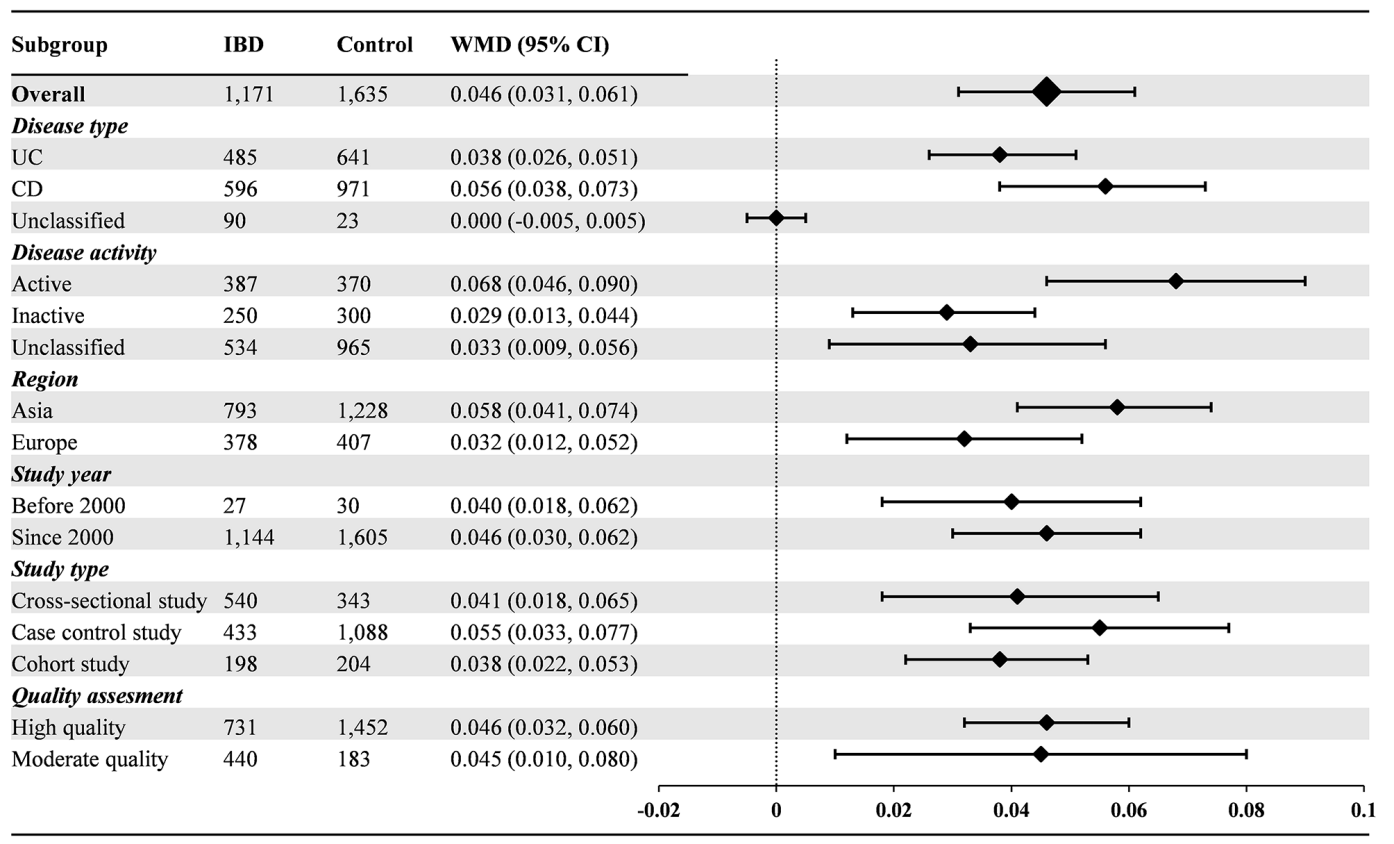
Weighted Mean Difference in PCT between the IBD Group and the Control Group. PCT measurements are reported in percentage (%). Abbreviations: IBD: inflammatory bowel disease; UC: ulcerative colitis; CD: Crohn’s disease; PCT: plateletcrit; WMD: weighted mean difference; 95% CI: 95% confidence interval

77 articles (161 available data) [20–45, 47–55, 57–98] reported the difference in PLT between the IBD group and the control group. The results indicated a significant increase in PLT values in IBD patients compared to healthy individuals (WMD: 69.910, 95% CI: 62.177, 77.643 10^9^/L, *P* < 0.001, Fig. 2). For MPV, the synthesis of data from 25 articles (with 55 available data) [23, 26, 31, 34, 36, 41, 45, 46, 49, 50, 52, 55, 56, 59, 60, 62, 68, 70, 77, 78, 84–86, 91, 92] revealed a significant decrease in MPV values in IBD patients when compared with healthy people (WMD: -0.912, 95% CI: -1.086, -0.739 fL, *P* < 0.001, Fig. 3). In the analysis of PDW, encompassing 8 articles (with 22 available data) [45, 60, 62, 63, 68, 85, 86, 97], no significant difference was observed between the IBD group and the control group (WMD: -0.207%, 95% CI: -0.655%, 0.241%, *P* = 0.260, Fig. 4). Turning to PCT, the inclusion of 9 articles (with 19 available data) [23, 34, 60, 62, 63, 68, 85, 86, 94] revealed a significant increase in PCT values among IBD patients compared to healthy people (WMD: 0.046%, 95% CI, 0.031%, 0.061%, *P* < 0.001, Fig. 5).

### Meta-regression analyses and planned subgroup analyses

The evidence demonstrated a high between-study heterogeneity (Figure S1-S4). Meta-regression analyses were performed based on covariates including disease type, disease activity, region, study year, study type, and quality assessment. The meta-regression results suggested that disease activity might serve as the primary source of heterogeneity among studies investigating abnormal platelet parameters in IBD (Table S5). Furthermore, study year potentially contributes to heterogeneity in PLT studies, while study type may be a source of heterogeneity in PDW studies. The remaining covariates did not exhibit significant heterogeneity in the studies of platelet parameters in IBD.

Furthermore, we conducted subgroup analyses based on disease type, disease activity, region, study year, study type, and quality assessment. As depicted in Fig. 2, both the UC group (WMD: 60.494, 95% CI: 45.298, 75.690 10^9^/L) and CD group (WMD: 82.952, 95% CI: 70.532, 95.372 10^9^/L) exhibited significantly higher PLT levels than healthy individuals. Regarding disease activity, PLT levels were significantly higher in both the active group (WMD: 95.451, 95% CI: 81.460, 109.442 10^9^/L) and inactive group (WMD: 21.866, 95% CI: 14.043, 29.688 10^9^/L) compared to the control group. In terms of different regions, PLT levels in the Asia group (WMD: 73.579, 95% CI: 59.348, 87.810 10^9^/L), Europe group (WMD: 68.076, 95% CI: 50.952, 85.200 10^9^/L), North America group (WMD: 93.697, 95% CI: 55.906, 131.487 10^9^/L), Africa group (WMD: 39.764, 95% CI: 6.294, 73.235 10^9^/L), and Oceania group (WMD: 25.752, 95% CI: 5.809, 45.696 10^9^/L) were all significantly higher than those in the control group. Among the subgroups in the study year, PLT levels were significantly higher in both the before 2000 group (WMD: 101.660, 95% CI: 66.770,136.550 10^9^/L) and the since 2000 group than in the control group (WMD: 66.611, 95% CI: 58.557, 77.466 10^9^/L). Concerning different study types, the cross-sectional study group (WMD: 70.932, 95% CI: 56.853, 85.011 10^9^/L), case control study group (WMD: 67.685, 95% CI: 55.484, 79.887 10^9^/L), and cohort study group (WMD: 68.116, 95% CI: 50.848, 85.384 10^9^/L) all had significantly higher PLT than healthy control. For studies of different quality scores, both the high quality group (WMD: 70.758, 95% CI: 62.545, 78.970 10^9^/L) and moderate quality group (WMD: 63.189, 95% CI: 37.400, 88.979 10^9^/L) demonstrated significantly higher PLT levels than healthy individuals.

For MPV, as illustrated in Fig. 3, MPV levels in both the UC group (WMD: -0.918, 95% CI: -1.218, -0.617 fL) and CD group (WMD: -1.026, 95% CI: -1.285, -0.767 fL) were significantly lower than healthy individuals, while the difference in the unclassified group was not statistically significant (WMD: -0.489, 95% CI: -0.980, 0.003 fL). Regarding disease activity, MPV levels were significantly lower in both the active group (WMD: -1.308, 95% CI: -1.640, -0.976 fL) and inactive group (WMD: -0.527, 95% CI: -0.958, -0.095) compared to the control group. Considering different regions, MPV levels in the Asia group (WMD: -1.103, 95% CI: -1.261, -0.787 fL), Europe group (WMD: -0.684, 95% CI: -0.882, -0.487 fL), and Africa group (WMD: -1.610, 95% CI: -1.990, -1.230 fL) were all significantly lower than those in the control group. In the study year subgroups, MPV levels were significantly lower in the since 2000 group than in the control group (WMD: -0.922, 95% CI: -1.092, -0.752 fL), while the difference was not statistically significant in the before 2000 group (WMD: -0.668, 95% CI: -1.393, 0.056 fL). Turning to different study types, the cross-sectional study group (WMD: -0.786, 95% CI: -1.034, -0.538 fL), case control study group (WMD: -1.090, 95% CI: -1.484, -0.695 fL), and cohort study group (WMD: -1.120, 95% CI: -1.394, -0.845 fL) all had significantly lower MPV than healthy control. In studies of different quality scores, both the high quality group (WMD: -0.966, 95% CI: -1.180, -0.752 fL) and moderate quality group (WMD: -0.678, 95% CI: -0.916, -0.440 fL) exhibited significantly lower MPV levels than healthy individuals.

As shown in Fig. 4, the difference in PDW between both the UC group (WMD: 0.444%, 95% CI: -0.284%, 1.173%) and CD group (WMD: -0.540%, 95% CI: -1.118%, 0.038%) was not statistically significant when compared with the control group. Regarding disease activity, PDW levels were significantly lower in the active group (WMD: -1.138%, 95% CI: -1.535%, -0.741%) than in the control group, while PDW levels were significantly higher in the unclassified group (WMD: 1.261%, 95% CI: 0.735%, 1.787%). There was no significant difference between inactive group (WMD: -0.161%, 95% CI: -0.640%, 0.317%) and the control group. Concerning different study types, the cross-sectional study group (WMD: -0.764%, 95% CI: -1.226%, -0.302%) and case control study group (WMD: -0.668%, 95% CI: -1.672%, -0.335%) had significantly lower PDW than healthy control, while the cohort study group had significantly higher PDW (WMD: 1.261%, 95% CI: 0.735%, 1.787%). For studies of different regions and quality scores, the difference in the Asia group (WMD: -0.076%, 95% CI: -0.707%, 0.555%) and Europe group (WMD: -0.283%, 95% CI: -0.798%, 0.231%), as well as the high quality group (WMD: -0.252%, 95% CI: -0.788%, 0.283%) and moderate quality group (WMD: -0.002%, 95% CI: -0.525%, 0.520%) was not significant compared with the healthy control.

As demonstrated in Fig. 5, both the UC group (WMD: 0.038%, 95% CI: 0.026%, 0.051%) and CD group (WMD: 0.056%, 95% CI: 0.038%, 0.073%) exhibited significantly higher PCT levels than healthy individuals, while the difference in the unclassified group was not statistically significant (WMD: 0.000%, 95% CI: -0.005%, 0.005%). Regarding disease activity, PCT levels were significantly higher in both the active group (WMD: 0.068%, 95% CI: 0.046%, 0.090%) and inactive group (WMD: 0.029%, 95% CI: 0.013%, 0.044%) compared to the control group. For studies of different regions, the PCT levels in the Aisa group (WMD: 0.058%, 95% CI: 0.041%, 0.074%) and Europe group (WMD: 0.032%, 95% CI: 0.012%, 0.052%) were significantly higher than those in the control group. Additionally, PCT levels were significantly higher in the before 2000 group (WMD: 0.040%, 95% CI: 0.018%, 0.062%) and since 2000 group (WMD: 0.046%, 95% CI: 0.030%, 0.062%), as well as the cross-sectional study group (WMD: 0.041%, 95% CI: 0.018%, 0.065%), case control study group (WMD: 0.055%, 95% CI: 0.033%, 0.077%), and cohort study group (WMD: 0.038%, 95% CI: 0.022%, 0.053%) when compared with the control group. In terms of study quality, both the high quality group (WMD: 0.046%, 95% CI: 0.032%, 0.060%) and moderate quality group (WMD: 0.045%, 95% CI: 0.010%, 0.080%) exhibited significantly higher PCT levels than those in the control group.

### Sensitivity analysis and publication bias

The sensitivity analysis revealed that individual studies did not exert a significant influence on the difference in PLT, MPV, PDW, and PCT between IBD patients and healthy people (Table S6). The funnel plots a general symmetry (Figure S2), and Egger’s test further indicated the absence of publication bias within the encompassed studies for PLT and PDW (Table S7, Figure S3). Nevertheless, publication bias was identified in studies in MPV and PCT (Table S7, Figure S3). Following the inclusion of an additional 13 and 10 studies, respectively, no substantial alterations were observed in the pooled effect values and 95% confidence intervals, signifying the stability of the results (Table S8, Figure S4).

## Discussion

This systematic review and meta-analysis reveals that PLT and PCT are significantly higher, while MPV is significantly lower in the IBD population compared with healthy people. However, no significant difference was observed in PDW between the two groups.

The initial evidence of a platelet abnormality in IBD was reported in 1968 when an increased PLT was observed in a case series of IBD patients having an exacerbation of clinical activity [99]. Regarding changes in PLT in IBD in this meta-analysis, PLT was significantly increased in both the UC and CD groups. The mechanisms underlying the abnormal increase in PLT in IBD patients remain elusive. Platelet production is mainly regulated by plasma thrombopoietin [100]. Under normal circumstances, platelet production is controlled by a negative feedback mechanism based on PLT in the blood, with platelet production being inversely correlated with thrombopoietin levels [101]. However, it is worth noting that the levels of thrombopoietin were significantly higher in patients with IBD than in the general population [102]. In addition, available data present ambiguity as other investigations have demonstrated a lack of correlation between PLT and thrombopoietin concentration [29], suggesting the existence of additional regulatory factors contributing to increased PLT in IBD [103]. Several studies have elucidated the pivotal role of platelet in connecting inflammation and coagulation in both UC and CD, establishing a detrimental cycle in which contributing parameters mutually propagate and intensify [8, 104]. There is compelling evidence supporting the notion that platelets function as potent proinflammatory cells, in addition to their role in hemostasis [105]. In IBD, platelet circulate in a highly activated state, as evidenced by an elevated concentration of circulating platelet activation markers in the systemic circulation of patients [106]. Specific platelet granular products, including P-selectin, GP IIb/IIIa, CD40L, and GP53, are assimilated into the cytoplasmic membrane, imparting them with a more adhesive and interactive phenotype [107]. Furthermore, during activation, platelets develop receptors for chemokines, cytokines, and complement components, enabling their participation in various inflammatory cascades in IBD [7]. In this meta-analysis, PLT levels were higher in both the active and inactive groups than in the control group, indicating a persistent state of activation in platelet and coagulation systems in IBD. It was demonstrated that, even without any stimulation, platelets from IBD patients displayed spontaneous aggregation and hypersensitivity [108]. This observation could potentially explain why one-third of thrombotic events occurred during clinical remission [109, 110]. PLT can serve as a simple marker for distinguishing between the active and inactive phases of IBD. The sensitivity and specificity of PLT in detecting active CD were reported at 80.0% and 86.7%, respectively, with an impressive identification accuracy reflected by an area under the receiver operating characteristic curve (AUROC) of 0.909 [85]. While elevated PLT is associated with IBD disease activity, it is not an independent predictor of thrombotic events in patients with IBD, as many other diseases, such as malignancies, can also lead to increased PLT [111].

For MPV, our meta-analysis found that the changes in MPV among IBD patients were significantly lower compared to those in the control group, irrespective of whether they belonged to the UC or CD groups or the active and inactive groups. A previous meta-analysis has reported a decrease in MPV in IBD patients [112], which was consistent with our findings. The reduced MPV levels were frequently reported and exhibited a correlation with endoscopic findings and disease activity indexes, such as C-reactive protein (CRP) and erythrocyte sedimentation rate (ESR) [68, 84]. Platelet volume decreases in the presence of an inflammatory process, primarily due to abnormalities in thrombopoiesis and increased platelet consumption. Inflammatory mediators stimulate bone marrow precursors to enhance platelet generation at the cost of maturation time, delivering smaller platelet in circulation, while at the same time larger and more active platelet are consumed at inflammatory sites [113, 114] Several studies have indicated that MPV could serve as a potential inflammatory marker for IBD. In a diagnostic study, MPV demonstrated a sensitivity of 60.0% and specificity of 77.5% for detecting active CD, while for active UC, the sensitivity and specificity were 56.3% and 77.5%, respectively. The AUROC was 0.718 for CD and 0.691 for UC [60]. However, Saler T. et al.’s studies contradicted these findings, suggesting that MPV lacked differential value in distinguishing between active and inactive IBD [115]. Further research is needed to determine whether MPV can reliably differentiate IBD activity.

PDW directly measures variability in platelet size, varies with platelet activity, and mirrors the heterogeneity in platelet morphology [116, 117]. Under physiological conditions, a direct correlation exists between MPV and PDW, with both typically changing in the same direction [117]. In our study, we found no statistical difference between the PDW levels in IBD patients and healthy people, but PDW levels in the active group were significantly lower than those in the normal control. Similar to our findings, previous studies have also found that patients with active IBD have significantly lower PDW levels than healthy controls [60, 68]. Moreover, patients with active IBD were found to have significantly lower PDW levels than those in remission [85]. PCT is a measurement method derived from PLT and MPV, and is numerically equal to the product of the two indicators. In the present study, PCT levels were significantly higher in the IBD population than in the healthy control group. Several other studies have also shown that PCT is significantly higher in IBD populations [60, 62, 63], with PCT demonstrating a significant association with ESR and white blood cell count [60]. It was also revealed that PDW was positively and PCT was negatively correlated with disease activity in UC [60]. In a diagnostic study by Galijašević M. et al. [85], the AUROC for the differentiation of UC and CD activity by PDW was 0.722 and 0.798, respectively, indicating fair diagnostic accuracy. However, this accuracy was surpassed by PCT, whose AUROC for the differentiation of UC and CD activity was 0.731 and 0.891, respectively. Tang J. et al.’s study demonstrated that PCT emerged as the most effective index for monitoring disease activity in CD patients with high-sensitivity CRP levels lower than 10.0 mg/L (sensitivity 71%, specificity 85%, AUROC 0.77) [68]. Nevertheless, it is noteworthy that PDW and PCT were often underestimated, and the number of studies conducted on these platelet parameters was far less than those on PLT and MPV.

The multifaceted nature of platelets positions them prominently in human health and various diseases, extending beyond IBD [118]. This meta-analysis represents a pioneering effort to systematically evaluate differences in all major platelet parameters between IBD patients and healthy individuals. We implemented a robust search strategy to identify relevant studies and examined differences in all significant platelet parameters between IBD patients and healthy individuals. Despite the novelty of our work, certain limitations should be noted. For instance, there was considerable heterogeneity in partial summary results. While subgroup analysis elucidated some sources of heterogeneity, others remained unexplained. In some studies, details regarding disease type and activity were lacking, and insufficient data extraction for additional confounders (e.g., sex, age, IBD medication) hindered further analysis. Furthermore, variations in the criteria for diagnosing and clinically evaluating IBD, coupled with differences in the platelet analyzers utilized, might introduce potential biases and challenge the generalizability of these findings to a broader context.

## Conclusion

In conclusion, this meta-analysis reveals that individuals with IBD exhibit significantly elevated PLT and PCT, alongside a significant decrease in MPV compared to healthy individuals. Moreover, PDW demonstrates a significant reduction only in patients with active IBD compared to those in a healthy state. The platelet parameters can offer reliable insights for assessing the severity of IBD and understanding potential pathophysiological mechanisms. Monitoring the clinical manifestations of platelet abnormalities serves as a valuable means to obtain diagnostic and prognostic information. Conversely, proactive measures should be taken to prevent the consequences of platelet abnormalities in individuals with IBD. Furthermore, there is a pressing requirement for more rigorous clinical trials to establish diagnostic criteria and advance the clinical evaluation of IBD at the platelet parameters level.

### Electronic supplementary material

Below is the link to the electronic supplementary material.

Supplementary Material 1

## Data Availability

The data that support the findings of this study are available from the corresponding author upon reasonable request.
