# Supplementary material for: Abnormal platelet parameters in inflammatory bowel disease: a systematic review and meta-analysis
Source: BMC Gastroenterol. 2024 Jul 3;24:214. doi: 10.1186/s12876-024-03305-9 (PMC11221001; doi:10.1186/s12876-024-03305-9)
Supplement: Supplementary file 1 — Supplementary Material 1 [file 12876_2024_3305_MOESM1_ESM.pdf]

Abnormal Platelet Parameters in Inflammatory Bowel Disease: A Systematic Review and Meta-Analysis

Supplementary Material

Supplementary Methods 1. The Newcastle Ottawa Quality Assessment Scale - Case control study
Supplementary Methods 2. The Newcastle Ottawa Quality Assessment Scale - Cohort study
Supplementary Methods 3. The Agency for Healthcare Research and Quality methodology checklist
Supplementary Table S1. The Preferred Reporting Items for a Systematic Review and Meta-analysis (PRISMA) guideline
Supplementary Table S2. The Meta-analysis of Observational Studies in Epidemiology (MOOSE) reporting guideline
Supplementary Table S3. Search Strategy
Supplementary Table S4. Characteristics of Included Studies
Supplementary Table S5. Meta-regression analysis
Supplementary Table S6. Sensitivity analysis
Supplementary Table S7. Egger's Test
Supplementary Table S8. The Trim and Fill Method
Supplementary Figure S1. Forest plot of the difference of platelet parameters between the IBD group and the control group.
A. PLT, B. MPV, C. PDW, D. PCT
Supplementary Figure S2. Funnel plot of the difference of platelet parameters between the IBD group and the control group.
A. PLT, B. MPV, C. PDW, D. PCT
Supplementary Figure S3. Egger's publication bias plot of the difference of platelet parameters between the IBD group and the control group.
A. PLT, B. MPV, C. PDW, D. PCT
Supplementary Figure S4. Filled funnel plot of the difference of MPV and PCT between the IBD group and the control group.
A. MPV, B. PCT

Supplementary Methods 1. The Newcastle Ottawa Quality Assessment Scale - Case control study

Note: A study can be awarded a maximum of one star for each numbered item within the Selection and Exposure categories. A maximum of two stars can be given for Comparability.

Selection
1) Is the case definition adequate?
a) yes, with independent validation ☆
b) yes, eg record linkage or based on self reports
c) no description
2) Representativeness of the cases
a) consecutive or obviously representative series of cases ☆
b) potential for selection biases or not stated
3) Selection of Controls
a) community controls ☆
b) hospital controls
c) no description
4) Definition of Controls
a) no history of disease (endpoint) ☆
b) no description of source
Comparability
1) Comparability of cases and controls on the basis of the design or analysis
a) study controls for _______________ (Select the most important factor.) ☆
b) study controls for any additional factor (This criteria could be modified to indicate specific control for a second important factor.) ☆

Exposure
1) Ascertainment of exposure
a) secure record (eg surgical records) ☆
b) structured interview where blind to case/control status ☆
c) interview not blinded to case/control status
d) written self report or medical record only
e) no description
2) Same method of ascertainment for cases and controls
a) yes ☆
b) no
3) Non-Response rate
a) same rate for both groups ☆
b) non respondents described
c) rate different and no designation

Supplementary Methods 2. The Newcastle Ottawa Quality Assessment Scale - Cohort study

Note: A study can be awarded a maximum of one star for each numbered item within the Selection and Outcome categories. A maximum of two stars can be given for Comparability

Selection

1) Representativeness of the exposed cohort

a) truly representative of the average _______________ (describe) in the community ☆

b) somewhat representative of the average ______________ in the community ☆

c) selected group of users eg nurses, volunteers

d) no description of the derivation of the cohort

2) Selection of the non-exposed cohort

a) drawn from the same community as the exposed cohort ☆

b) drawn from a different source

c) no description of the derivation of the non-exposed cohort

3) Ascertainment of exposure

a) secure record (eg surgical records) ☆

b) structured interview ☆

c) written self-report

d) no description

4) Demonstration that outcome of interest was not present at start of study

a) yes ☆

b) no

Comparability

1) Comparability of cohorts on the basis of the design or analysis

a) study controls for _____________ (select the most important factor) ☆

b) study controls for any additional factor (This criteria could be modified to indicate specific control for a second important factor.) ☆

Outcome

1) Assessment of outcome

a) independent blind assessment ☆

b) record linkage ☆

c) self-report

d) no description

2) Was follow-up long enough for outcomes to occur

a) yes (select an adequate follow up period for outcome of interest) ☆

b) no

3) Adequacy of follow up of cohorts

a) complete follow up - all subjects accounted for ☆

b) subjects lost to follow up unlikely to introduce bias - small number lost - > ____ % (select an adequate %) follow up, or description provided of those lost) ☆

c) follow up rate < ____% (select an adequate %) and no description of those lost

d) no statement

Supplementary Methods 3. The Agency for Healthcare Research and Quality methodology checklist

Item

1) Define the source of information (survey, record review)

2) List inclusion and exclusion criteria for exposed and unexposed subjects (cases and controls) or refer to previous publications

3) Indicate time period used for identifying patients

4) Indicate whether or not subjects were consecutive if not population-based

5) Indicate if evaluators of subjective components of study were masked to other aspects of the status of the participants

6) Describe any assessments undertaken for quality assurance purposes (e.g., test/retest of primary outcome measurements)

7) Explain any patient exclusions from analysis

8) Describe how confounding was assessed and/or controlled.

9) If applicable, explain how missing data were handled in the analysis

10) Summarize patient response rates and completeness of data collection

11) Clarify what follow-up, if any, was expected and the percentage of patients for which incomplete data or follow-up was obtained

Supplementary Table S1. The Preferred Reporting Items for a Systematic Review and Meta-analysis (PRISMA) guideline

Section and Topic	Item #	Checklist item	Location where item is reported
**TITLE**			
Title	1	Identify the report as a systematic review.	Title
**ABSTRACT**			
Abstract	2	See the PRISMA 2020 for Abstracts checklist.	Abstract
**INTRODUCTION**			
Rationale	3	Describe the rationale for the review in the context of existing knowledge.	Introduction, Paragraph 1, 2
Objectives	4	Provide an explicit statement of the objective(s) or question(s) the review addresses.	Introduction, Paragraph 3
**METHODS**			
Eligibility criteria	5	Specify the inclusion and exclusion criteria for the review and how studies were grouped for the syntheses.	Methods, Paragraph 3, 4, 7
Information sources	6	Specify all databases, registers, websites, organisations, reference lists and other sources searched or consulted to identify studies. Specify the date when each source was last searched or consulted.	Methods, Paragraph 2
Search strategy	7	Present the full search strategies for all databases, registers and websites, including any filters and limits used.	Table S3
Selection process	8	Specify the methods used to decide whether a study met the inclusion criteria of the review, including how many reviewers screened each record and each report retrieved, whether they worked independently, and if applicable, details of automation tools used in the process.	Methods, Paragraph 5
Data collection process	9	Specify the methods used to collect data from reports, including how many reviewers collected data from each report, whether they worked independently, any processes for obtaining or confirming data from study investigators, and if applicable, details of automation tools used in the process.	Methods, Paragraph 5
Data items	10a	List and define all outcomes for which data were sought. Specify whether all results that were compatible with each outcome domain in each study were sought (e.g. for all measures, time points, analyses), and if not, the methods used to decide which results to collect.	Methods, Paragraph 5
	10b	List and define all other variables for which data were sought (e.g. participant and intervention characteristics, funding sources). Describe any assumptions made about any missing or unclear information.	Methods, Paragraph 5
Study risk of bias assessment	11	Specify the methods used to assess risk of bias in the included studies, including details of the tool(s) used, how many reviewers assessed each study and whether they worked independently, and if applicable, details of automation tools used in the process.	Methods, Paragraph 5, 6
Effect measures	12	Specify for each outcome the effect measure(s) (e.g. risk ratio, mean difference) used in the synthesis or presentation of results.	Methods, Paragraph 7
Synthesis methods	13a	Describe the processes used to decide which studies were eligible for each synthesis (e.g. tabulating the study intervention characteristics and comparing against the planned groups for each synthesis (item #5)).	Methods, Paragraph 3, 4, 7
	13b	Describe any methods required to prepare the data for presentation or synthesis, such as handling of missing summary statistics, or data conversions.	Methods, Paragraph 7
	13c	Describe any methods used to tabulate or visually display results of individual studies and syntheses.	Methods, Paragraph 7
	13d	Describe any methods used to synthesize results and provide a rationale for the choice(s). If meta-analysis was performed, describe the model(s), method(s) to identify the presence and extent of statistical heterogeneity, and software package(s) used.	Methods, Paragraph 7, 8, 9, 10
	13e	Describe any methods used to explore possible causes of heterogeneity among study results (e.g. subgroup analysis, meta-regression).	Methods,

Section and Topic	Item #	Checklist item	Location where item is reported
			Paragraph 8
	13f	Describe any sensitivity analyses conducted to assess robustness of the synthesized results.	Methods, Paragraph 9
Reporting bias assessment	14	Describe any methods used to assess risk of bias due to missing results in a synthesis (arising from reporting biases).	Methods, Paragraph 9
Certainty assessment	15	Describe any methods used to assess certainty (or confidence) in the body of evidence for an outcome.	Methods, Paragraph 7
**RESULTS**			
Study selection	16a	Describe the results of the search and selection process, from the number of records identified in the search to the number of studies included in the review, ideally using a flow diagram.	Results, Paragraph 1, Figure 1
	16b	Cite studies that might appear to meet the inclusion criteria, but which were excluded, and explain why they were excluded.	Figure 1
Study characteristics	17	Cite each included study and present its characteristics.	Results, Paragraph 2, Table S4
Risk of bias in studies	18	Present assessments of risk of bias for each included study.	Results, Paragraph 2, Table S4
Results of individual studies	19	For all outcomes, present, for each study: (a) summary statistics for each group (where appropriate) and (b) an effect estimate and its precision (e.g. confidence/credible interval), ideally using structured tables or plots.	Results, Paragraph 3, 5, 6, 7, 8 Figure 2, 3, 4, 5 Figure S1, S2, S3, S4
Results of syntheses	20a	For each synthesis, briefly summarise the characteristics and risk of bias among contributing studies.	Results, Paragraph 2, Table S4
	20b	Present results of all statistical syntheses conducted. If meta-analysis was done, present for each the summary estimate and its precision (e.g. confidence/credible interval) and measures of statistical heterogeneity. If comparing groups, describe the direction of the effect.	Results, Paragraph 3, 5, 6, 7, 8
	20c	Present results of all investigations of possible causes of heterogeneity among study results.	Results, Paragraph 4
	20d	Present results of all sensitivity analyses conducted to assess the robustness of the synthesized results.	Results, Paragraph 9
Reporting biases	21	Present assessments of risk of bias due to missing results (arising from reporting biases) for each synthesis assessed.	NA
Certainty of evidence	22	Present assessments of certainty (or confidence) in the body of evidence for each outcome assessed.	Results, Paragraph 3, 5, 6, 7, 8 Figure 2, 3, 4, 5 Figure S1, S2, S3, S4
**DISCUSSION**			
Discussion	23a	Provide a general interpretation of the results in the context of other evidence.	Discussion, Paragraph 1, 2, 3, 4

Section and Topic	Item #	Checklist item	Location where item is reported
	23b	Discuss any limitations of the evidence included in the review.	Discussion, Paragraph 5
	23c	Discuss any limitations of the review processes used.	Discussion, Paragraph 5
	23d	Discuss implications of the results for practice, policy, and future research.	Conclusion, Paragraph 1
**OTHER INFORMATION**			
Registration and protocol	24a	Provide registration information for the review, including register name and registration number, or state that the review was not registered.	Methods, Paragraph 1
	24b	Indicate where the review protocol can be accessed, or state that a protocol was not prepared.	Methods, Paragraph 1
	24c	Describe and explain any amendments to information provided at registration or in the protocol.	NA
Support	25	Describe sources of financial or non-financial support for the review, and the role of the funders or sponsors in the review.	Funding
Competing interests	26	Declare any competing interests of review authors.	Competing interests
Availability of data, code and other materials	27	Report which of the following are publicly available and where they can be found: template data collection forms; data extracted from included studies; data used for all analyses; analytic code; any other materials used in the review.	Data availability

Abbreviations: NA, not apply

Supplementary Table S2. The Meta-analysis of Observational Studies in Epidemiology (MOOSE) reporting guideline

Item No	Recommendation	Location where item is reported
**Reporting of background should include**		
1	Problem definition	Introduction, Paragraph 3
2	Hypothesis statement	Introduction, Paragraph 2
3	Description of study outcome(s)	Introduction, Paragraph 3
4	Type of exposure or intervention used	NA
5	Type of study designs used	Methods, Paragraph 3
6	Study population	Introduction, Paragraph 1, 2, 3
**Reporting of search strategy should include**		
7	Qualifications of searchers (eg, librarians and investigators)	Methods, Paragraph 2, 5
8	Search strategy, including time period included in the synthesis and keywords	Methods, Paragraph 2, Table S3
9	Effort to include all available studies, including contact with authors	Methods, Paragraph 5
10	Databases and registries searched	Methods, Paragraph 1, 2
11	Search software used, name and version, including special features used (eg, explosion)	NA
12	Use of hand searching (eg, reference lists of obtained articles)	Methods, Paragraph 2
13	List of citations located and those excluded, including justification	Figure 1
14	Method of addressing articles published in languages other than English	NA
15	Method of handling abstracts and unpublished studies	NA
16	Description of any contact with authors	Methods, Paragraph 5
**Reporting of methods should include**		
17	Description of relevance or appropriateness of studies assembled for assessing the hypothesis to be tested	Methods, Paragraph 5
18	Rationale for the selection and coding of data (eg, sound clinical principles or convenience)	Methods, Paragraph 5
19	Documentation of how data were classified and coded (eg, mutiple raters, blinding, and interrater reliability)	Methods, Paragraph 5
20	Assessment of confounding (eg, comparability of cases and controls in studies where appropriate)	Methods, Paragraph 6, 8

Item No	Recommendation	Location where item is reported
21	Assessment of study quality, indluding blinding of quality assessors; stratifcation or regression on possible predictors of study results	Methods, Paragraph 6
22	Assessment of heterogeneity	Methods, Paragraph 8
23	Description of statistical methods (eg, complete description of fixed or random effects models, justification of whether the chosen models account for predictors of study results, dose-response models, or cumulative meta-analysis) in sufficient detail to be replicated	Methods, Paragraph 7, 8, 9
24	Provision of appropriate tables and graphics	Figure 1, 2, 3, 4, 5
**Reporting of results should include**		
25	Graphic summarizing individual study estimates and overall estimate	Figure 2 ,3, 4, 5; Figure S1, S2, S3, S4
26	Table giving descriptive information for each study included	Table S4
27	Results of sensitivity testing (eg, subgroup analysis)	Results, Paragraph 4, 5, 6, 7, 8, 9
28	Indication of statistical uncertainty of findings	Results, Paragraph 9
**Reporting of discussion should include**		
29	Quantitative assessment of bias (eg, publication bias)	Results, Paragraph 2, 9
30	Justification for exclusion (eg, exclusion of non-English-language citations)	Figure 1
31	Assessment of quality of included studies	Results, Paragraph 2
**Reporting of conclusions should include**		
32	Consideration of alternative explanations for observed results	Discussion, paragraph 2, 3, 4<br>Conclusion
33	Generalization of the conclusions (ie, appropriate for the data presented and within the domain of the literature review)	Conclusion
34	Guidelines for future research	Conclusion
35	Disclosure of funding source	Funding

Abbreviations: NA, not apply

Supplementary Table S3. Search Strategy

Database	Search strategy		Results
**1)PubMed (To December 16, 2023)**	#1	"Inflammatory Bowel Diseases"[MeSH Terms]	98,693
	#2	"inflammatory bowel disease*"[Title/Abstract] OR "crohn*"[Title/Abstract] OR "ulcerative colitis*"[Title/Abstract] OR "IBD"[Title/Abstract]	129,265
	#3	#1 OR #2	142,747
	#4	"Mean Platelet Volume "[MeSH Terms] OR "Platelet Count" [MeSH Terms] OR "platelet distribution width" [Title/Abstract] OR "plateletcrit" [Title/Abstract]	25,683
	#5	"mean platelet volume*"[Title/Abstract] OR "platelet volume, mean"[Title/Abstract] OR "volume mean platelet"[Title/Abstract] OR "MPV"[Title/Abstract] OR "count platelet"[Title/Abstract] OR "platelet count*"[Title/Abstract] OR "platelet number*"[Title/Abstract] OR "number platelet"[Title/Abstract] OR "blood platelet number*"[Title/Abstract] OR ("number*" AND "blood platelet"[Title/Abstract]) OR "platelet number blood"[Title/Abstract] OR "blood platelet count*"[Title/Abstract] OR "count blood platelet"[Title/Abstract] OR "platelet count blood"[Title/Abstract] OR "PLT"[Title/Abstract] OR "PDW"[Title/Abstract] OR "PCT"[Title/Abstract]	61,121
	#6	#4 OR #5	72,209
	#7	#3 AND #6	483
**2) Embase (To December 16, 2023)**	#1	'inflammatory bowel diseases'/exp	208,681
	#2	'inflammatory bowel disease*':ti,ab,kw OR crohn*:ti,ab,kw OR 'ulcerative colitis*':ti,ab,kw OR ibd:ti,ab,kw	216,003
	#3	#1 OR #2	253,296
	#4	'mean platelet volume'/exp OR 'platelet count'/exp OR 'platelet distribution width'/exp OR 'plateletcrit'/exp	134,415
	#5	'mean platelet volume*':ti,ab,kw OR 'platelet volume*, mean':ti,ab,kw OR 'volume*, mean platelet':ti,ab,kw OR mpv:ti,ab,kw OR 'count*, platelet':ti,ab,kw OR 'platelet count*':ti,ab,kw OR 'platelet number*':ti,ab,kw OR 'number*, platelet[':ti,ab,kw OR 'blood platelet number*':ti,ab,kw OR 'number*, blood platelet':ti,ab,kw OR 'platelet number*, blood':ti,ab,kw OR 'blood platelet count*':ti,ab,kw OR 'count*, blood platelet':ti,ab,kw OR 'platelet count*, blood':ti,ab,kw OR plt:ti,ab,kw OR pdw:ti,ab,kw OR pct:ti,ab,kw	109,371
	#6	#4 OR #5	179,975
	#7	#3 AND #6	2,102

Database	Search strategy		Results
**3) Cochrane Library (To December 16, 2023)**	#1	MeSH descriptor: [Inflammatory Bowel Diseases] explode all trees	4,897
	#2	(inflammatory bowel disease* OR crohn* OR ulcerative colitis* OR IBD):ti,ab,kw	13,201
	#3	#1 or #2	13,201
	#4	MeSH descriptor: [Mean Platelet Volume] explode all trees	28
	#5	MeSH descriptor: [Platelet Count] explode all trees	2,123
	#6	(Platelet Distribution Width OR Plateletcrit OR Mean Platelet Volume*OR Platelet Volume*, Mean OR Volume*, Mean Platelet OR MPV OR Count*, Platelet OR Platelet Count* OR Platelet Number* OR Number*, Platelet OR Blood Platelet Number* OR Number*, Blood Platelet OR Platelet Number*, Blood OR Blood Platelet Count* OR Count*, Blood Platelet OR Platelet Count*, Blood OR PLT OR PDW blood OR PLT OR PDW OR PCT):ti,ab,kw	18,424
	#7	#4 OR #5 OR #6	18,425
	#8	#3 AND #7	145
**4) Web of science (To December 16, 2023)**	#1	TS= ("inflammatory bowel disease*" OR "crohn*" OR "ulcerative colitis*" OR "IBD")	321,440
	#2	TS= ("Mean Platelet Volume*" OR "Platelet Volume*, Mean" OR "Volume*, Mean Platelet" OR MPV OR "Platelet Count*" OR "Count*, Platelet" OR "Platelet Number*" OR "Number*, Platelet" OR "Blood Platelet Number*" OR "Number*, Blood Platelet" OR "Platelet Number*, Blood" OR "Blood Platelet Count*" OR "Count*, Blood Platelet" OR "Platelet Count*, Blood" OR PLT OR "platelet distribution width" OR PDW OR Plateletcrit OR PCT)	142,811
	#3	#1 AND #2	1,375

Supplementary Table S4. Characteristics of Included Studies

Source	Study type	Disease type	Case group							Control group							Activity assessment	Hematology Analyzer	NOS	AHRQ Score
			Sample (n)	Gender (M/F)	Age (year)	PLT (10^9/L)	MPV (fL)	PDW (%)	PCT (%)	Sample (n)	Gender (M/F)	Age (year)	PLT (10^9/L)	MPV (fL)	PDW (%)	PCT (%)				
Lam A, et al, 1975, Canada (20)	Case control study	UC	12	5/7	45.25±17.35	341±71.4	NA	NA	NA	15	NA	30.23±8.91[b]	194±11.5	NA	NA	NA	NA	Coulter Counter Model F	7	NA
		CD	12	4/8	31.17±12.53	282±33.4	NA	NA	NA	15	NA	30.23±8.91[b]	194±11.5	NA	NA	NA	NA	Coulter Counter Model F	7	NA
Bygdeman S, et al, 1977, Sweden (21)	Case control study	CD	20	NA	39.5±15.2	333±22	NA	NA	NA	20	NA	37.5±14.1	327±22	NA	NA	NA	NA	Ljungberg Celloscope 202	7	NA
Fukushima K, et al,1990, Japan (22)	Cross-sectional study	UC	17	9/8	31.68±7.79[b]	340±125	NA	NA	NA	623	300/323	37.66±6.34	258±59	NA	NA	NA	TWSI	Counter Model S-Plus VI	NA	7
		CD	18	10/8	30.8±10.16[b]	368±158	NA	NA	NA	623	300/323	37.66±6.34	258±59	NA	NA	NA	IOIBD assessment	Counter Model S-Plus VI	NA	7
Zhao L, et al, 1990, China (23)	Case control study	Active UC	27	20/7	NA	301.10±80.37	7.53±1.65	NA	0.22±0.05	30	NA	NA	211.30±38.90	8.71±1.08	NA	0.18±0.03	TWSI	Baker-810	7	NA
Wiedermann CJ, et al, 1994, Austria (24)	Case control study	UC	22	9/13	39.8±3.45	318.1±30.52	NA	NA	NA	8	3/5	57.0±6.21	210.7±13.75	NA	NA	NA	TWSI	NA	8	NA
		CD	20	8/12	33.3±2.89	421.8±36.67	NA	NA	NA	8	3/5	57.0±6.21	210.7±13.75	NA	NA	NA	(DeDombal FT, et al, 1974)	NA	8	NA
Chiarantini E, 1996, Turkey (25)	Case control study	Active UC	19	9/10	46.5±15.4	307±74	NA	NA	NA	40	22/18	39.5±11.4	225±32	NA	NA	NA	modified TWSI	Coulter LH 780	8	NA
		CD	41	24/17	37.2±12.7	351±137	NA	NA	NA	40	22/18	39.5±11.4	225±32	NA	NA	NA	CDAI	Coulter LH 780	8	NA

Source	Study type	Disease type	Case group							Control group							Activity assessment	Hematology Analyzer	NOS	AHRQ Score
		Active CD	8	NA	NA	352±130.2	NA	NA	NA	40	22/18	39.5±11.4	225±32	NA	NA	NA	CDAI	Coulter LH 780	8	NA
		Inactive CD	33	NA	NA	323±137	NA	NA	NA	40	22/18	39.5±11.4	225±32	NA	NA	NA	CDAI	Coulter LH 780	8	NA
Järemo P, 1996, Sweden (26)	Case control study	Active IBD	18	NA	NA	314.94±68.65	6.71±0.47	NA	NA	12	NA	NA	275.42±72.63	7.63±0.83	NA	NA	NA	NA	6	NA
		Inactive IBD	9	NA	NA	286.89±71.64	7.67±0.48	NA	NA	12	NA	NA	275.42±72.63	7.63±0.83	NA	NA	NA	NA	6	NA
Chiarantini E, et al, 1997, Italy (27)	Cohort study	CD	31	17/14	33.8±11	357±117	NA	NA	NA	30	NA	NA	224±39	NA	NA	NA	CDAI	NA	7	NA
Heits F, et al, 1999, Germany (28)	Cross-sectional study	Active IBD	30	37/22	31.69±13.01[b]	448.45±147.04[b]	NA	NA	NA	56	28/28	31.64±8.09[b]	246.39±47.67[b]	NA	NA	NA	Rachmilewitz index / CDAI	NA	NA	8
		Inactive IBD	29		31.69±13.01[b]	316.91±76.49[b]	NA	NA	NA	56	28/28	31.64±8.09[b]	246.39±47.67[b]	NA	NA	NA	Rachmilewitz index / CDAI	NA	NA	8
Kapsoritakis A, et al, 2000, Greece (29)	Case control study	Active UC	35	41/22	52.85±13.7[b]	275±101	NA	NA	NA	42	24/18	37.8±8.3[b]	248±55	NA	NA	NA	SCCAI	Cell-Dyn 3200 system	8	NA
		Inactive UC	28			237±96	NA	NA	NA	42	24/18	37.8±8.3[b]	248±55	NA	NA	NA	SCCAI	Cell-Dyn 3200 system	8	NA
		Active CD	22	25/15	41.04±13.7[b]	376±128	NA	NA	NA	42	24/18	37.8±8.3[b]	248±55	NA	NA	NA	CDAI	Cell-Dyn 3200 system	8	NA
		Inactive CD	18			245±85	NA	NA	NA	42	24/18	37.8±8.3[b]	248±55	NA	NA	NA	CDAI	Cell-Dyn 3200 system	8	NA
Sturm A, et al, 2000, Germany (30)	Case control study	UC	29	13/16	37.2±15.2	335±129	NA	NA	NA	28	15/13	31.8±9.1	276±85	NA	NA	NA	TWSI	NA	8	NA
		CD	45	27/18	36.1±11.6	336±92	NA	NA	NA	28	15/13	31.8±9.1	276±85	NA	NA	NA	CDAI	NA	8	NA

Source	Study type	Disease type	Case group							Control group							Activity assessment	Hematology Analyzer	NOS	AHRQ Score
Kapsoritakis A, et al, 2001, Greece (31)	Case control study	Active UC	54	60/33	49.5±12.9[b]	272±97	8.5±0.9	NA	NA	38	21/17	37.8±8.4[b]	251±56	9.4±1.2	NA	NA	SCCAI	Cell-Dyn 3200 system	8	NA
		Inactive UC	39			232±89	9.0±1.1	NA	NA	38	21/17	37.8±8.4[b]	251±56	9.4±1.2	NA	NA	SCCAI	Cell-Dyn 3200 system	8	NA
		Active CD	40	41/25	43.3±12.6[b]	356 ±123	7.8±1.0	NA	NA	38	21/17	37.8±8.4[b]	251±56	9.4±1.2	NA	NA	CDAI	Cell-Dyn 3200 system	8	NA
		Inactive CD	26			247±86	8.9±1.3	NA	NA	38	21/17	37.8±8.4[b]	251±56	9.4±1.2	NA	NA	CDAI	Cell-Dyn 3200 system	8	NA
van Bodegraven AA, et al, 2001, Netherlands (32)	Cohort study	Active UC (before treatment)	33	21/12	39.3±14.7[b]	313±96.0[c]	NA	NA	NA	22	10/12	36.4±7.3[b]	238±46.0[c]	NA	NA	NA	(van der Heide H, et al, 1988)	NA	6	NA
		Inactive UC (after treatment)	30	19/11	NA	273±65.8[c]	NA	NA	NA	22	10/12	36.4±7.3[b]	238±46.0[c]	NA	NA	NA	(van der Heide H, et al, 1988)	NA	6	NA
Dai X, et al, 2003, China (33)	Cross-sectional study	Active CD	13	8/5	31±12.0[b]	288±54	NA	NA	NA	10	5/5	35±12.9[b]	150±42	NA	NA	NA	CDAI	NA	NA	10
		Inactive CD	10	6/4	28±10.3[b]	200±46	NA	NA	NA	10	5/5	35±12.9[b]	150±42	NA	NA	NA	CDAI	NA	NA	10
Kapsoritakis A, et al, 2003, Greece (34)	Cross-sectional study	IBD	90	NA	NA	278±117	8.4±0.1	NA	0.23±0.01	23	NA	37.7±9.3[b]	243±109	9.4±0.2	NA	0.23±0.01	Clinical Colitis Activity Index & CDAI	NA	NA	7
Dong WG, et al, 2004, China (35)	Cohort study	Active UC	39	16/23	46.4±8.4[b]	201.8±48.6	NA	NA	NA	30	19/11	NA	158.2±32.5	NA	NA	NA	(Jones SC, et al, 1994)	NA	8	NA
		Inactive UC	25	11/14	NA	173.7±36.4	NA	NA	NA	30	19/11	NA	158.2±32.5	NA	NA	NA	(Jones SC, et al, 1994)	NA	8	NA

Source	Study type	Disease type	Case group							Control group							Activity assessment	Hematology Analyzer	NOS	AHRQ Score
Irving P, et al, 2004, UK (36)	Cross-sectional study	IBD	67	27/40	33.12±7.33[b]	325.2±123.5[a]	8.2±1.0[a]	NA	NA	20	10/10	35.6±7.8[b]	243.3±34.3[a]	8.6±1.0[a]	NA	NA	HBI & SCCAI	ADVIA®120	NA	8
Andoh A, et al, 2006, Japan (37)	Cross-sectional study	UC	24	13/11	35.6 ± 15.5	333 ± 150	NA	NA	NA	22	12/10	33.4 ± 7.5	192 ± 33	NA	NA	NA	Rachmilewitz index	NA	NA	7
		CD	25	12/13	32.4 ± 7.5	287 ± 96	NA	NA	NA	22	12/10	33.4 ± 7.5	192 ± 33	NA	NA	NA	CDAI	NA	NA	7
Drzewoski J, et al 2006, Poland (38)	Cross-sectional study	Active UC	15	17/13	50.3±14.7	395±84	NA	NA	NA	21	12/9	53.1±12.8	225±60	NA	NA	NA	(Schroeder KW, et al, 1987)	NA	NA	8
		Inactive UC	15			267±69	NA	NA	NA	21	12/9	53.1±12.8	225±60	NA	NA	NA	(Schroeder KW, et al, 1987)	NA	NA	8
Efrat B, et al, 2006, Israel (39)	Cross-sectional study	CD	30	21/9	24.3 ± 12.1	315 ± 92.1	NA	NA	NA	28	15/13	36.8 ± 12.6	214.2 ± 59.3	NA	NA	NA	HBI	Coulter SKS instrument	NA	8
Payzin B, et al, 2006, Turkey (40)	Cross-sectional study	Active IBD	39	NA	36.2 ± 11.7[b]	415.8±52.2	NA	NA	NA	37	15/22	35.2 ± 7.1[b]	251.7 ± 65.4	NA	NA	NA	NA	NA	NA	7
		Inactive IBD	8	NA		232.6±66.8	NA	NA	NA	37	15/22	35.2 ± 7.1[b]	251.7 ± 65.4	NA	NA	NA	NA	NA	NA	7
Kayahan H, et al, 2007, Turkey (41)	Cross-sectional study	Active UC	16	NA	51.62 ± 18.36	366.4 ± 130.1	7.91 ± 0.9	NA	NA	20	NA	NA	228.0±34.8	9.18 ± 1.02	NA	NA	TWSI	FACSCalibur flow cytometer	NA	8
		Inactive UC	21	NA	47.85 ± 11.83	254.0 ± 59.3	8.77 ± 0.92	NA	NA	20	NA	NA	228.0±34.8	9.18 ± 1.02	NA	NA	TWSI	FACSCalibur flow cytometer	NA	8
Maher MM, et al, 2008, Egypt (42)	Cross-sectional study	UC	19	8/11	36±4.9[b]	271±98	NA	NA	NA	40	23/17	34±6.0	231±22	NA	NA	NA	TWSI	Cell-Dyn 3200 system	NA	8
		CD	7	4/3	32±8.1[b]	382±130	NA	NA	NA	40	23/17	34±6.0	231±22	NA	NA	NA	CDAI	Cell-Dyn 3200 system	NA	8

Source	Study type	Disease type	Case group							Control group							Activity assessment	Hematology Analyzer	NOS	AHRQ Score
Cakal B, et al, 2009, Turkey (43)	Case control study	Active UC	43	50/24	39.8±11.8	412±151	NA	NA	NA	20	7/13	37.6±8.0	326.1±66.5	NA	NA	NA	TWSI	NA	8	NA
		Inactive UC	31			332±92.8	NA	NA	NA	20	7/13	37.6±8.0	326.1±66.5	NA	NA	NA	TWSI	NA	8	NA
		Active CD	14	13/9	37.2±10.6	439±171	NA	NA	NA	20	7/13	37.6±8.0	326.1±66.5	NA	NA	NA	CDAI	NA	8	NA
		Inactive CD	8			282±86	NA	NA	NA	20	7/13	37.6±8.0	326.1±66.5	NA	NA	NA	CDAI	NA	8	NA
Krzystek-Korpacka M, et al, 2009, Poland (44)	Cohort study	Active UC	27	15/12	39.6±14.8[b]	382±151.7[c]	NA	NA	NA	81	48/33	33.8±7.6[b]	247±124.0[c]	NA	NA	NA	SCCAI	NA	7	NA
		Inactive UC	42	25/17	46.1±13.8[b]	267±88.2[c]	NA	NA	NA	81	48/33	33.8±7.6[b]	247±124.0[c]	NA	NA	NA	SCCAI	NA	7	NA
		Active CD	42	20/22	37.5±14.0[b]	383±174.9[c]	NA	NA	NA	81	48/33	33.8±7.6[b]	247±124.0[c]	NA	NA	NA	CDAI	NA	7	NA
		Inactive CD	13	6/7	37.1±12.9[b]	262±107.6[c]	NA	NA	NA	81	48/33	33.8±7.6[b]	247±124.0[c]	NA	NA	NA	CDAI	NA	7	NA
Shen J, et al, 2009, China (45)	Cohort study	CD Male	50	50/26	45.39±14.16	253.20±107.51	9.52±1.92	14.49±3.45	NA	74	74/42	43.74±15.57	193.36±46.87	10.74±1.03	12.89±2.26	NA	SCCAI	Abbott Cell-Dyn 3700 system	9	NA
		CD Female	26			275.88±118.28	9.73±2.75	14.18±3.5	NA	42			183.83±47.58	11.20±1.08	13.44±2.39	NA	SCCAI	Abbott Cell-Dyn 3700 system	9	NA
		UC Male	93	93/102	46.39±15.66	224.24±96.43	9.83±1.95	15.28±3.47	NA	74			193.36±46.87	10.74±1.03	12.89±2.26	NA	Baron score	Abbott Cell-Dyn 3700 system	9	NA
		UC Female	102			246.15±95.43	10.83±10.13	15.76±2.69	NA	42			183.83±47.58	11.20±1.08	13.44±2.39	NA	Baron score	Abbott Cell-Dyn 3700 system	9	NA
Yüksel O, et al, 2009, Turkey (46)	Cross-sectional study	Active UC	37	41/20	42.18±12.17	NA	8.06±1.19	NA	NA	27	18/9	38.22±10.77	NA	8.65±0.79	NA	NA	Rachmilewitz index	Beckman Coulter analyzer	NA	8

Source	Study type	Disease type	Case group							Control group							Activity assessment	Hematology Analyzer	NOS	AHRQ Score
		Inactive UC	24	41/20	42.18±12.17	NA	8.45±0.87	NA	NA	27	18/9	38.22±10.77	NA	8.65±0.79	NA	NA	Rachmilewitz index	Beckman Coulter analyzer	NA	8
Krzystek-Korpacka M, et al, 2010, Poland (47)	Cross-sectional study	Active UC	42	22/20	42.9±14.0[b]	345±176.5[c]	NA	NA	NA	105	66/39	38.6±8.9[b]	266±85.4[c]	NA	NA	NA	Mayo scores	NA	NA	9
		Inactive UC	51	31/20	49.7±13.3[b]	274±67.6[c]	NA	NA	NA	105	66/39	38.6±8.9[b]	266±85.4[c]	NA	NA	NA	Mayo scores	NA	NA	9
		Active CD	53	24/29	41.5±10.6[b]	389±268.5[c]	NA	NA	NA	105	66/39	38.6±8.9[b]	266±85.4[c]	NA	NA	NA	CDAI	NA	NA	9
		Inactive CD	28	12/16	41.7±12.4[b]	249±203.7[c]	NA	NA	NA	105	66/39	38.6±8.9[b]	266±85.4[c]	NA	NA	NA	CDAI	NA	NA	9
Krzystek-Korpacka M, et al, 2010, Poland (48)	Cross-sectional study	Active CD	57	31/26	41.1±10.5[b]	375±178.5[c]	NA	NA	NA	108	64/44	41.1±8.9[b]	241±151.2[c]	NA	NA	NA	CDAI	NA	NA	9
		Inactive CD	34	20/14	39.9±12.0[b]	278±110.3[c]	NA	NA	NA	108	64/44	41.1±8.9[b]	241±151.2[c]	NA	NA	NA	CDAI	NA	NA	9
Arhan M, et al, 2011, Turkey (49)	Case control study	UC	105	59/46	39.7±10.7	311.66±97.35	8.5±1.0	NA	NA	43	31/12	35.3±10.2	269.21±87.19	8.9±0.6	NA	NA	Endoscopic activity index	Coulter STKS	9	NA
		CD	60	39/21	37.7±13.2	349.10±111.71	8.0±1.0	NA	NA	43	31/12	35.3±10.2	269.21±87.19	8.9±0.6	NA	NA	CDAI	Coulter STKS	9	NA
Dogan Y, et al, 2011, Turkey (50)	Cross-sectional study	IBD	69	NA	39.69±13.21	291.67±78.73	8.51±1.34	NA	NA	38	NA	41.59±13.12	266.39±55.23	8.56±0.83	NA	NA	TWSI & CDAI	Beckman Coulter-LH 780	NA	9
Mohammadi M, et al, 2011, Iran (51)	Cross-sectional study	Active UC	37	19/18	39.46±17.066	267.54±139.759	NA	NA	NA	20	11/9	35.4±13.9	266.430±6.89	NA	NA	NA	criteria of American Gastroenterology Association	sysmex Kx21n	NA	7

Source	Study type	Disease type	Case group							Control group							Activity assessment	Hematology Analyzer	NOS	AHRQ Score
		Inactive UC	23	7/16	34.00±13.921	227.33±77.051	NA	NA	NA	20	11/9	35.4±13.9	266.430±6.89	NA	NA	NA	criteria of American Gastroenterology Association	sysmex Kx21n	NA	7
Polińska B, et al, 2011, Poland (52)	Cross-sectional study	UC	16	9/7	NA	300.56±110.96	8.03±0.83	NA	NA	32	11/21	NA	248±44.74	8.68±0.64	NA	NA	TWSI	Bayer Advia 120	NA	8
Yarur AJ, et al, 2011, USA (53)	Cohort study	IBD	356	172/184	44.62±15.82	339.1±115.69	NA	NA	NA	712	344/268	45.11±16.02	278.3±73.32	NA	NA	NA	NA	NA	9	NA
Yeşil A, et al, 2011, Turkey (54)	Cross-sectional study	Active UC	35	26/35	40.3±12	386±52	NA	NA	NA	44	21/23	38.9±11	249±45	NA	NA	NA	TWSI	NA	NA	9
		Inactive UC	26			271±67	NA	NA	NA	44	21/23	38.9±11	249±45	NA	NA	NA	TWSI	NA	NA	9
		Active CD	29	30/26	38±10	327±64	NA	NA	NA	44	21/23	38.9±11	249±45	NA	NA	NA	CDAI	NA	NA	9
		Inactive CD	27			262±55	NA	NA	NA	44	21/23	38.9±11	249±45	NA	NA	NA	CDAI	NA	NA	9
Huang Q, et al, 2012, China (55)	Case control study	Active UC	65	54/42	38.7±7.4	278.3±123.6	9.80±1.60	NA	NA	60	34/26	38.8±8.6	154.5±60.2	13.32±1.90	NA	NA	Southerland disease activity index	sysmex XE-2100	8	NA
		Inactive UC	31			201.3±97.5	10.63±1.70	NA	NA	60	34/26	38.8±8.6	154.5±60.2	13.32±1.90	NA	NA	Southerland disease activity index	sysmex XE-2100	8	NA
Liu S, et al, 2012, China (56)	Cohort study	Active CD	29	40/21	32.4±1.59	NA	9.52±0.223	NA	NA	50	35/15	34.0±1.44	NA	11.1±0.160	NA	NA	CDAI	NA	9	NA
		Inactive CD	32			NA	9.58±0.251	NA	NA	50	35/15	34.0±1.44	NA	11.1±0.160	NA	NA	CDAI	NA	9	NA
Akdoğan RA, et al, 2013, Turkey (57)	Cross-sectional study	UC	37	21/16	48±15	309±72	NA	NA	NA	30	9/21	45±8	278±65	NA	NA	NA	TWSI	Beckman Coulter Immage 800	NA	9

Source	Study type	Disease type	Case group							Control group							Activity assessment	Hematology Analyzer	NOS	AHRQ Score
Garg M, et al, 2013, Australia (58)	Cross-sectional study	UC	31	17/14	44±14.6[b]	248±81.3[b]	NA	NA	NA	23	10/13	39±11.9[b]	229±53.9[b]	NA	NA	NA	SCCCAI	NA	NA	9
		CD	40	22/18	41±12.3[b]	252±87.9[b]	NA	NA	NA	23	10/13	39±11.9[b]	229±53.9[b]	NA	NA	NA	HBI	NA	NA	9
Liu W, et al, 2013, China (59)	Cross-sectional study	Active UC (mild)	17	45/75	38.2±17.5	181.25±35.70	11.76±2.71	NA	NA	90	56/34	46.7±24.1	157.23±15.54	11.97±1.75	NA	NA	Mayo scores	NA	NA	8
		Active UC (moderate)	33			246.49±51.22	10.37±1.75	NA	NA	90	56/34	46.7±24.1	157.23±15.54	11.97±1.75	NA	NA	Mayo scores	NA	NA	8
		Active UC (severe)	27			320.15±84.31	8.32±0.96	NA	NA	90	56/34	46.7±24.1	157.23±15.54	11.97±1.75	NA	NA	Mayo scores	NA	NA	8
		Inactive UC	43			169.53±32.75	11.89±1.54	NA	NA	90	56/34	46.7±24.1	157.23±15.54	11.97±1.75	NA	NA	Mayo scores	NA	NA	8
		Active CD (moderate)	24	33/45	33.9±16.0	238.81±31.59	9.77±1.01	NA	NA	90	56/34	46.7±24.1	157.23±15.54	11.97±1.75	NA	NA	CDAI	NA	NA	8
		Active CD (severe)	15			326.01±44.31	8.21±1.66	NA	NA	90	56/34	46.7±24.1	157.23±15.54	11.97±1.75	NA	NA	CDAI	NA	NA	8
		Inactive CD	39			167.36±21.14	11.42±2.03	NA	NA	90	56/34	46.7±24.1	157.23±15.54	11.97±1.75	NA	NA	CDAI	NA	NA	8
Öztürk Z, et al, 2013, Turkey (60)	Cross-sectional study	Active UC	103	54/49	37.69±13.32	360.69±116.52	8.38±1.24	15.28±2.60	0.31±0.10	40	23/17	34.67±10.03	259.38±55.60	8.98±0.98	15.98±1.30	0.23±0.05	TWSI	Cell-Dyn 3700 SL analyzer	NA	6
		Inactive UC	103	54/49	37.69±13.32	315.19±116.77	8.16±1.20	16.26±1.77	0.27±0.07	40	23/17	34.67±10.03	259.38±55.60	8.98±0.98	15.98±1.30	0.23±0.05	TWSI	Cell-Dyn 3700 SL analyzer	NA	6
		Active CD	72	29/43	37.74±12.30	389.80±134.17	8.23±1.32	15.75±2.14	0.30±0.09	40	23/17	34.67±10.03	259.38±55.60	8.98±0.98	15.98±1.30	0.23±0.05	CDAI	Cell-Dyn 3700 SL analyzer	NA	6
		Inactive CD	72	29/43	37.74±12.30	329.47±94.12	8.30±1.44	16.49±1.11	0.27±0.07	40	23/17	34.67±10.03	259.38±55.60	8.98±0.98	15.98±1.30	0.23±0.05	CDAI	Cell-Dyn 3700 SL analyzer	NA	6
Schoepfer AM, et al, 2013, Switzerland (61)	Cross-sectional study	UC	228	138/90	41±13	370±132	NA	NA	NA	52	NA	NA	281±79	NA	NA	NA	modified Baron Index	NA	NA	9

Source	Study type	Disease type	Case group							Control group							Activity assessment	Hematology Analyzer	NOS	AHRQ Score
Voudoukis E, et al, 2013, Greece (62)	Cohort study	UC	91	33/58	48±19.6[a]	288.4±91.1[a]	8.4±1.1[a]	16.9±0.9[a]	0.2374±0.0640[a]	102	NA	NA	218.3±36.1[a]	9.5±1.0[a]	16.4±0.6[a]	0.2079±0.0368[a]	SCCCAI	CELL-DYN 3700	7	NA
		CD	107	50/57	40.3±15.6[a]	297.4±109.0[a]	8.6±1.3[a]	17.1±1.0[a]	0.2532±0.0646[a]	102	NA	NA	218.3±36.1[a]	9.5±1.0[a]	16.4±0.6[a]	0.2079±0.0368[a]	CDAI	CELL-DYN 3700	7	NA
Avdagic N, et al, 2014, Bosnia and Herzegovina (63)	Case control study	Active CD	12	14/16	34.7±12.3[a]	353.27±59.12[a]	NA	17.11±0.88[a]	0.285±0.040[a]	30	13/17	38.6±12.2[a]	253.23±8.81	NA	17.54±0.90[a]	0.231±0.008	(van Hees PA, et al, 1980)	Siemens Healthcare Diagnosis automatic hematology analyzer	8	NA
		Inactive CD	18			286.22±64.60[a]	NA	17.77±0.98[a]	0.254±0.065[a]	30	13/17	38.6±12.2[a]	253.23±8.81	NA	17.54±0.90[a]	0.231±0.008	(van Hees PA, et al, 1980)	Siemens Healthcare Diagnosis automatic hematology analyzer	8	NA
Ciećko-Michalska I, et al, 2014, Turkey (64)	Cross-sectional study	UC	32	16/16	32.7±9.0[b]	382.78±73.32	NA	NA	NA	30	17/13	33.6±6.6[b]	230.50±44.62	NA	NA	NA	Mayo scores	NA	NA	8
		CD	31	18/13	32.5±7.1[b]	349±109.14	NA	NA	NA	30	17/13	33.6±6.6[b]	230.50±44.62	NA	NA	NA	CDAI	NA	NA	8
Dolapcioglu C, et al, 2014, Turkey (65)	Cross-sectional study	UC	33	20/13	46.8±6.76	438±15.19	NA	NA	NA	26	10/16	36.2±5.47	247±16.97	NA	NA	NA	TWSI	NA	NA	8
		CD	18	7/11	29.4±4.83	510±22.58	NA	NA	NA	26	10/16	36.2±5.47	247±16.97	NA	NA	NA	CDAI	NA	NA	8
Huo H, et al. 2014, China (66)	Cross-sectional study	Active UC	100	52/48	46.1±10.5	463.7±121.4	NA	NA	NA	100	51/49	46.6±10.8	174.7±24.3	NA	NA	NA	NA	NA	NA	8

Source	Study type	Disease type	Case group							Control group							Activity assessment	Hematology Analyzer	NOS	AHRQ Score
		Inactive UC	100	50/50	45.9±11.3	177.4±25.9	NA	NA	NA	100	51/49	46.6±10.8	174.7±24.3	NA	NA	NA	NA	NA	NA	8
Garg M, et al, 2015, Australia (67)	Cross-sectional study	UC	15	6/9	42±10.9[b]	258±69.3[b]	NA	NA	NA	19	11/8	38±12.2[b]	226±56.3[b]	NA	NA	NA	HBI	NA	NA	8
		CD	19	11/8	45±14.4[b]	262±96.2[b]	NA	NA	NA	19	11/8	38±12.2[b]	226±56.3[b]	NA	NA	NA	SCCAI	NA	NA	8
Tang J, et al, 2015, China (68)	Case control study	Active CD	103	62/68	33.4 ± 3.2	350.49 ± 127.13	9.92 ± 0.93	10.46 ± 1.65	0.34 ± 0.11	130	67/63	31.6 ± 1.2	192.70 ± 34.80	10.44 ± 0.89	12.21 ± 1.72	0.22 ± 0.04	CDAI	NA	8	NA
		Inactive CD	27			287.30 ± 106.92	9.88 ± 1.19	11.52 ± 2.69	0.28 ± 0.10	130	67/63	31.6 ± 1.2	192.70 ± 34.80	10.44 ± 0.89	12.21 ± 1.72	0.22 ± 0.04	CDAI	NA	8	NA
Cibor D, et al, 2017, Poland (69)	Cross-sectional study	Active UC	26	22/25	36.6±16.1[a]	318.49 ±99.67[a]	NA	NA	NA	50	35/15	37.1±13.0[a]	232.33 ±52.67[a]	NA	NA	NA	(Schroeder KW, et al, 1987)	NA	9	NA
		Inactive UC	21			266.96 ±69.17[a]	NA	NA	NA	50	35/15	37.1±13.0[a]	232.33 ±52.67[a]	NA	NA	NA	(Schroeder KW, et al, 1987)	NA	9	NA
		Active CD	22	20/18	28.4±9.2[a]	333.83±76.87[a]	NA	NA	NA	50	35/15	37.1±13.0[a]	232.33 ±52.67[a]	NA	NA	NA	CDAI	NA	9	NA
		Inactive CD	16			277.74 ±103.23[a]	NA	NA	NA	50	35/15	37.1±13.0[a]	232.33 ±52.67[a]	NA	NA	NA	CDAI	NA	9	NA
Gawrońska B, et al, 2017, Poland (70)	Cross-sectional study	UC	32	18/14	NA	304.43±114.46	8.30±1.08	NA	NA	32	15/17	NA	247.78±44.74	8.67±0.63	NA	NA	TWSI	ADVIA 2120	NA	6
Szczeklik K, et al, 2017, Poland (71)	Cross-sectional study	CD	62	33/29	30.5±2.1	421.6±36.2	NA	NA	NA	40	22/18	32.1±2.8	234.8±11.0	NA	NA	NA	CDAI	Sysmex XE-2100	NA	10
Ye L, et al, 2017, China (72)	Cross-sectional study	Active UC	35	25/20	38.00±13.85	279.45±98.17	NA	NA	NA	30	15/15	36.47 ± 6.39	236.53±59.94	NA	NA	NA	Mayo scores	NA	NA	8

Source	Study type	Disease type	Case group							Control group							Activity assessment	Hematology Analyzer	NOS	AHRQ Score
		Inactive UC	10	25/20	38.00±13.85	239.88±187.50	NA	NA	NA	30	15/15	36.47 ± 6.39	236.53±59.94	NA	NA	NA	Mayo scores	NA	NA	8
		Active CD	26	30/15	36.07±11.40	271.29±108.63	NA	NA	NA	30	15/15	36.47 ± 6.39	236.53±59.94	NA	NA	NA	CDAI	NA	NA	8
		Inactive CD	19			197.56±78.50	NA	NA	NA	30	15/15	36.47 ± 6.39	236.53±59.94	NA	NA	NA	CDAI	NA	NA	8
Chen G, et al, 2018, China (73)	Cross-sectional study	CD	60	40/20	33.57±10.02	244±48.15	NA	NA	NA	60	35/25	35.64±11.58	152±30.01	NA	NA	NA	NA	SYSMEX XE-2100	NA	9
Szczeklik K, et al, 2018, Poland (74)	Cross-sectional study	Active CD	32	19/13	34.6±11.3	364.4±173.9[a]	NA	NA	NA	26	14/12	33.8±9.6	257.1±57.6[a]	NA	NA	NA	CDAI	NA	NA	8
		Inactive CD	27	17/9	36.1±15.1	263.7±36.3[a]	NA	NA	NA	26	14/12	33.8±9.6	257.1±57.6[a]	NA	NA	NA	CDAI	NA	NA	8
Szczeklik K, et al, 2018, Poland (75)	Cross-sectional study	CD	58	32/26	35.6±12.69	313.5±116.54	NA	NA	NA	25	12/13	34.16±9.84	258.72±45.29	NA	NA	NA	CDAI	NA	NA	6
Wang D, et al, 2018, China (76)	Cross-sectional study	Active UC	63	32/31	53.87±10.99	277.3±99.0	NA	NA	NA	53	28/25	52.56±10.85	235.2±56.3	NA	NA	NA	Mayo scores	NA	NA	9
		Inactive UC	22	12/10	52.94±11.64	256.4±62.9	NA	NA	NA	53	28/25	52.56±10.85	235.2±56.3	NA	NA	NA	Mayo scores	NA	NA	9
Coşkun Y, 2019, Turkey (77)	Cross-sectional study	Active UC	83	53/30	43.23±12.63	309.6±115.7	8.35±0.89	NA	NA	20	8/12	38.5±10.65	272.5±72.2	9.04±0.74	NA	NA	TWSI	NA	NA	9
		Inactive UC	83	53/30	43.23±12.63	268±88	8.59±0.8	NA	NA	20	8/12	38.5±10.65	272.5±72.2	9.04±0.74	NA	NA	TWSI	NA	NA	9
Erecan E, et al, 2019, Turkey (78)	Cross-sectional study	UC	40	16/24	48.25±16.25	282.55±94.58	18.57±1.92	NA	NA	40	21/19	40.52±13.56	272.66±70.77	18.92±2.17	NA	NA	NA	Cobas autoanalyzer	NA	8
Jiang Q, et al, 2019, China (79)	Case control study	Active CD	108	68/40	35.71±14.48	286.73±24.82	NA	NA	NA	55	30/25	45.09±3.40	241.58±14.45	NA	NA	NA	CDAI	ACL-TOP-700	8	NA
Kothari HG, et al, 2019,	Cross-sectional	Inactive UC	83	47/36	37.06±14.87	343±133	NA	NA	NA	42	25/17	37.17±11.96	276±78	NA	NA	NA	Mayo scores	NA	NA	9

Source	Study type	Disease type	Case group							Control group							Activity assessment	Hematology Analyzer	NOS	AHRQ Score
India (80)	study																			
Bai X, et al, 2020, Russia (81)	Cross-sectional study	Active UC	50	25/25	39±9	350±6	NA	NA	NA	50	24/26	40±10	220±5	NA	NA	NA	disease activity index	BS-220	NA	9
Li L, et al, 2020, China (82)	Case control study	UC	69	38/31	48.9±15.6	267.29±109.34	NA	NA	NA	138	76/62	49.1±15.9	209.68±59.2	NA	NA	NA	Mayo scores	NA	9	NA
		CD	137	73/64	36.8±12.6	294.58±103.86	NA	NA	NA	274	146/128	35.7±11.6	208.97±50.39	NA	NA	NA	CDAI	NA	9	NA
Sari C, et al, 2020, Turkey (83)	Cross-sectional study	Active IBD	26	37/35	42.8 ± 13.4	340.4 ± 72.3	NA	NA	NA	93	44/49	37.0 ± 9.2	242.4±49.6	NA	NA	NA	Mayo scores / CDAI	NA	NA	10
		Inactive IBD	46		41.7 ± 13.4	267.8 ± 69 0	NA	NA	NA	93	44/49	37.0 ± 9.2	242.4±49.6	NA	NA	NA	Mayo scores / CDAI	NA	NA	10
Chen Z, et al, 2021, China (84)	Cross-sectional study	Active UC (mild)	43	72/60	49.42 ± 14.57	226.230±81.316	9.934±1.748	NA	NA	208	98/110	51.66 ± 10.10	219.850 ± 51.324	9.910 ± 1.620	NA	NA	Mayo scores	NA	NA	9
		Active UC (moderate)	74			278.790±114.070	8.969±1.418	NA	NA	208	98/110	51.66 ± 10.10	219.850 ± 51.324	9.910 ± 1.620	NA	NA	Mayo scores	NA	NA	9
		Active UC (severe)	10			346.100±84.125	8.343±1.691	NA	NA	208	98/110	51.66 ± 10.10	219.850 ± 51.324	9.910 ± 1.620	NA	NA	Mayo scores	NA	NA	9
		Inactive UC	5			242.400 ± 93.071	10.380 ± 0.268	NA	NA	208	98/110	51.66 ± 10.10	219.850 ± 51.324	9.910 ± 1.620	NA	NA	Mayo scores	NA	NA	9
Galijašević M, et al, 2021, Austria (85)	Cross-sectional study	Active UC	15	14/16	34.7±9.8	336.5±63.3	8.1±0.88	16.7±0.96	0.27±0.05	30	13/17	39.8±12.1	253.2±48.3	9.1±1.1	17.8±0.98	0.23±0.05	TWSI	NA	NA	8
		Inactive UC	15			278.7±50.4	8.7±0.81	17.6±0.91	0.24±0.04	30	13/17	39.8±12.1	253.2±48.3	9.1±1.1	17.8±0.98	0.23±0.05	TWSI	NA	NA	8
		Active CD	15	13/17	40.7±15.2	357.3±84.9	8.7±0.95	14.4±3.4	0.33±0.06	30	13/17	39.8±12.1	253.2±48.3	9.1±1.1	17.8±0.98	0.23±0.05	HBI	NA	NA	8
		Inactive CD	15			252.9±55.4	9.3±0.88	16.5±3.16	0.23±0.05	30	13/17	39.8±12.1	253.2±48.3	9.1±1.1	17.8±0.98	0.23±0.05	HBI	NA	NA	8

Source	Study type	Disease type	Case group							Control group							Activity assessment	Hematology Analyzer	NOS	AHRQ Score
Zhang G, et al, 2021, China (86)	Cross-sectional study	Active CD	40	30/10	37.15±14.08	280.82±83.67	10.46±1.00	12.17±2.22	0.28±0.07	40	23/17	33.48±5.67	239.43±63.73	11.42±1.61	13.31±2.49	0.24±0.07	NA	NA	NA	9
Zhang MH, et al, 2021, China (87)	Cross-sectional study	UC	172	91/81	46.60±15.45[a]	251.67±85.98[a]	NA	NA	NA	172	96/76	46.80±14.20[a]	214.58±53.45[a]	NA	NA	NA	Mayo scores	NA	NA	9
Ammar SR, et al, 2022, Egypt (88)	Cross-sectional study	UC	30	13/17	34.2±10.07	305.3±91.19	NA	NA	NA	30	18/12	32.6±11.46	307±80.84	NA	NA	NA	NA	Siemens ADVIA 2120	NA	9
Lu J, et al, 2022, China (89)	Cross-sectional study	CD	862	593/269	33±13	279.80±95.04[a]	NA	NA	NA	576	336/240	34±11	207.70±50.54[a]	NA	NA	NA	HBI	Beckman Coulter LH 780	NA	9
Abdel Hameed NA, et al, 2023, Egypt (90)	Case control study	UC	30	19/11	31.93±10.68	324.43±1.11	NA	NA	NA	30	14/16	29.13±6.97	299.27±1.75	NA	NA	NA	NA	NA	7	NA
		CD	34	21/13	33.12±11.94	376.85±1.77	NA	NA	NA	30	14/16	29.13±6.97	299.27±1.75	NA	NA	NA	CDAI	NA	7	NA
Basha OM, et al, 2023, Egypt (91)	Case control study	UC	48	28/20	36.63±11.01	385.7±67.6	8.61±1.08	NA	NA	48	24/24	32±9.25	383.3±62.6	10.22±0.8	NA	NA	TWSI	NA	7	NA
Feng R, et al, 2023, China (92)	Cohort study	CD	136	103/33	32.3±11.44	368.8±134.12	9.295±1.143	NA	NA	126	95/31	34.9±11.67	221.9±88.8	9.748±1.053	NA	NA	CDAI	NA	9	NA
Geng B, et al, 2023, China (93)	Case control study	Active UC (mild)	37	20/17	50.24±13.23	245.41±62.95	NA	NA	NA	90	48/42	50.06±10.09	224.19±46.28	NA	NA	NA	Mayo scores	NA	7	NA
		Active UC (moderate)	41	31/10	43.27±17.71	302.54±102.42	NA	NA	NA	90	48/42	50.06±10.09	224.19±46.28	NA	NA	NA	Mayo scores	NA	7	NA
		Active UC (severe)	38	18/20	47.11±17.54	362.76±110.03	NA	NA	NA	90	48/42	50.06±10.09	224.19±46.28	NA	NA	NA	Mayo scores	NA	7	NA
Huang J, et al, 2023, China (94)	Case control study	UC	131	73/58	43.89±19.49[a]	266.76±84.71[a]	NA	NA	0.2165±0.0525[a]	369	237/132	48.75±26.05[a]	197.82±42.42[a]	NA	NA	0.1830±0.0298[a]	Mayo scores	Coulter 780 5 Diff analyzer	8	NA
		CD	115	76/39	46.19±18.02[a]	312.03±118.62[a]	NA	NA	0.2470±0.0751[a]	369	237/132	48.75±26.05[a]	197.82±42.42[a]	NA	NA	0.1830±0.0298[a]	Simple Endoscopic Scores for CD	Coulter 780 5 Diff analyzer	8	NA

Source	Study type	Disease type	Case group							Control group							Activity assessment	Hematology Analyzer	NOS	AHRQ Score
Liu Q, et al, 2023, China (95)	Cross-sectional study	Active CD	30	20/10	26.05±12.26[a]	271.64±107.81[a]	NA	NA	NA	15	10/5	32.90±3.04	184.80±13.78	NA	NA	NA	CDAI	NA	NA	9
Sleutjes JAM, et al, 2023, The Netherlands (96)	Cross-sectional study	IBD	235	103/132	58.05±11.19[a]	291±65	NA	NA	NA	829	365/464	61.35±8.17[a]	266±62	NA	NA	NA	SCCAI / HBI	NA	NA	10
Xue J, et al, 2023, China (97)	Cross-sectional study	Active CD (mild)	106	76/30	29.3±13.2	325.6±117.1	NA	10.53±1.90	NA	293	190/103	30.1±12.3	230.10±51.08	NA	11.89±1.75	NA	CDAI	NA	NA	10
		Active CD (moderate to severe)	88	61/27	27.9±10.7	354.9±121.6	NA	10.46±1.67	NA	293	190/103	30.1±12.3	230.10±51.08	NA	11.89±1.75	NA	CDAI	NA	NA	10
		Inactive CD	109	79/30	28.6±11.0	288.3±102.1	NA	11.04±2.02	NA	293	190/103	30.1±12.3	230.10±51.08	NA	11.89±1.75	NA	CDAI	NA	NA	10
Ye Y, et al, 2023, China (98)	Cross-sectional study	Active UC (mild)	15	8/7	52.20±11.08	266.88±74.32	NA	NA	NA	15	7/8	41.30±14.71	240.10±63.37	NA	NA	NA	Mayo scores	NA	NA	9
		Active UC (moderate)	15	8/7	43.90±11.72	285.86±122.68	NA	NA	NA	15	7/8	41.30±14.71	240.10±63.37	NA	NA	NA	Mayo scores	NA	NA	9
		Active UC (severe)	15	7/8	40.80±15.72	347.09±110.97	NA	NA	NA	15	7/8	41.30±14.71	240.10±63.37	NA	NA	NA	Mayo scores	NA	NA	9

Abbreviations: UC, ulcerative colitis; CD, Crohn's disease; IBD, Inflammatory bowel disease; M, male; F, Female; PLT, platelet count; MPV, mean platelet volume; PDW, platelet distribution width; PCT, plateletcrit; NA, not applicable; NOS: Newcastle-Ottawa Scale; AHRQ: Agency for Healthcare Research and Quality TWSI, Truelove and Witts severity index; CDAI, Crohn's disease activity index; SCCAI, simple clinical colitis activity index; HBI, Harvey-Bradshaw Index

Values are presented as mean±SD;

[a]: Converted value, the original value was presented in median (25–75th percentile)

[b]: Converted value, the original value was presented in median (range)

[c]: Converted value, the original value was presented in mean (95% CI)

Supplementary Table S5. Meta-regression analysis

	Variable	Coeff.	Std. Err.	t	*P*	95% CI	Adj R-squared
**PLT**	***Disease type***						3.66%
	UC	8.972688	18.36243	0.49	0.626	(-27.29481, 45.24018)	
	CD	32.79348	18.57636	1.77	0.079	(-3.89653, 69.48349)	
	Unclassified	Ref.	Ref.	Ref.	Ref.	Ref.	
	Disease activity						26.95%
	Active	15.17817	9.399698	1.61	0.108	(-3.3871, 33.74344)	
	Inactive	-58.79865	10.22608	-5.75	**<0.001**	(-78.9961, -38.60121)	
	Unclassified	Ref.	Ref.	Ref.	Ref.	Ref.	
	Region						0.59%
	Asia	-23.10719	33.18508	-0.7	0.487	(-88.65726, 42.44288)	
	Europe	-28.73108	33.56178	-0.86	0.393	(-95.02524, 37.56307)	
	North America	Ref.	Ref.	Ref.	Ref.	Ref.	
	Africa	-54.29393	40.52392	-1.34	0.182	(-134.3403, 25.75246)	
	Oceania	-69.5076	43.9103	-1.58	0.115	(-156.2431, 17.22787)	
	Study Year						2.83%
	Before 2000	35.2692	15.06832	2.34	**0.02**	(5.509332, 65.02907)	
	Since 2000	Ref.	Ref.	Ref.	Ref.	Ref.	
	Study type						-1.31%
	Cross-sectional study	1.101619	15.34863	0.07	0.943	(-29.21334, 31.41657)	
	Case control study	-2.2769	16.65013	-0.14	0.891	(-35.16243, 30.60863)	
	Cohort study	Ref.	Ref.	Ref.	Ref.	Ref.	
	Quality assesment						-0.49%
	High quality	7.608645	14.51469	0.52	0.601	(-21.0578, 36.27509)	
	Moderate quality	Ref.	Ref.	Ref.	Ref.	Ref.	
**MPV**	***Disease type***						-0.79%
	UC	-0.442079	0.409171	-1.08	0.285	(-1.263141, 0.3789832)	
	CD	-0.568759	0.426192	-1.33	0.188	(-1.423976, 0.2864584)	
	Unclassified	Ref.	Ref.	Ref.	Ref.	Ref.	
	Disease activity						15.03%
	Active	-0.7823066	0.261166	-3	**0.004**	(-1.306374, -0.2582395)	
	Inactive	Ref.	Ref.	Ref.	Ref.	Ref.	
	Unclassified	-0.2417272	0.283203	-0.85	0.397	(-0.8100147, 0.3265603)	
	Region						1.22%
	Asia	0.5920773	0.839948	0.7	0.484	(-1.093402, 2.277557)	
	Europe	0.9574346	0.854626	1.12	0.268	(-0.7574979, 2.672367)	
	Africa	Ref.	Ref.	Ref.	Ref.	Ref.	
	Study year						-1.66%
	Before 2000	Ref.	Ref.	Ref.	Ref.	Ref.	
	Since 2000	0.2578217	0.520039	0.5	0.622	(-0.785245, 1.300888)	
	Study type						-0.51%
	Cross-sectional study	0.3039455	0.330334	0.92	0.362	(-0.3589186, 0.9668096)	

	Case control study	0.0012321	0.37141	0	0.997	(-0.7440562, 0.7465204)	
	Cohort study	Ref.	Ref.	Ref.	Ref.	Ref.	
	Quality assesment						-0.17%
	High quality	-0.3259751	0.322899	-1.01	0.317	(-0.9736288, 0.3216786)	
	Moderate quality	Ref.	Ref.	Ref.	Ref.	Ref.	
**PDW**	***Disease type***						11.01%
	UC	Ref.	Ref.	Ref.	Ref.	Ref.	
	CD	-1.002468	0.557423	-1.8	0.087	(-2.165233, 0.1602957)	
	Disease activity						74.45%
	Active	-2.436718	0.392776	-6.2	**<0.001**	(-3.258808, -1.614629)	
	Inactive	-1.461215	0.415451	-3.52	**0.002**	(-2.330763, -0.5916676)	
	Unclassified	Ref.	Ref.	Ref.	Ref.	Ref.	
	Region						-5.86%
	Asia	0.3991948	0.586465	0.68	0.504	(-0.8241497, 1.622539)	
	Europe	Ref.	Ref.	Ref.	Ref.	Ref.	
	Study type						58.71%
	Cross-sectional study	-0.0983491	0.498024	-0.2	0.846	(-1.140725, 0.9440266)	
	Case control study	Ref.	Ref.	Ref.	Ref.	Ref.	
	Cohort study	1.999112	0.563484	3.55	**0.002**	(0.8197267, 3.178497)	
	Quality assesment						-5.86%
	High quality	Ref.	Ref.	Ref.	Ref.	Ref.	
	Moderate quality	0.2270263	0.721465	0.31	0.756	(-1.277924, 1.731976)	
**PCT**	***Disease type***	-9.02×10-7	0.0257072	0.00	1.000	(-0.0544968, 0.0544968)	19.80%
	UC	0.0388016	0.0278	1.4	0.182	(-0.0201321, 0.0977354)	
	CD	0.0555488	0.027156	2.05	0.058	(-0.0020182, 0.1131159)	
	Unclassified	Ref.	Ref.	Ref.	Ref.	Ref.	
	Disease activity						35.90%
	Active	0.0355692	0.013948	2.55	**0.021**	(0.0060017, 0.0651367)	
	Inactive	-0.0039287	0.015053	-0.26	0.797	(-0.0358389, 0.0279815)	
	Unclassified	Ref.	Ref.	Ref.	Ref.	Ref.	
	Region						17.73%
	Asia	0.0254939	0.013089	1.95	0.068	(-0.0021221, 0.05311)	
	Europe	Ref.	Ref.	Ref.	Ref.	Ref.	
	Study year						-6.98%
	Before 2000	Ref.	Ref.	Ref.	Ref.	Ref.	
	Since 2000	0.006127	0.031956	0.19	0.85	(-0.0612938, 0.0735477)	
	Study type						-6.40%
	Cross-sectional study	-0.0144892	0.015583	-0.93	0.366	(-0.0475228, 0.0185445)	
	Case control study	Ref.	Ref.	Ref.	Ref.	Ref.	
	Cohort study	-0.0178725	0.024284	-0.74	0.472	(-0.0693524, 0.0336073)	
	Quality assesment						-6.88%
	High quality	0.0021411	0.016228	0.13	0.897	(-0.0320968, 0.0363789)	
	Moderate quality	Ref.	Ref.	Ref.	Ref.	Ref.	

Abbreviations: UC, ulcerative colitis; CD, Crohn's disease; PLT, platelet count; MPV, mean platelet volume; PDW, platelet distribution width; PCT, plateletcrit; 95% CI: 95% confidence interval; Ref., reference

Supplementary Table S6. Sensitivity analysis

	Study omitted	Estimate	95% CI
**PLT**			
	Lam A (1975)	69.437809	(61.683231, 77.192387)
	Lam A (1975)	69.782303	(62.018652, 77.545954)
	Bygdeman S (1977)	70.370611	(62.607122, 78.1341)
	FEuropeushima K (1990)	69.847617	(62.094063, 77.601171)
	FEuropeushima K (1990)	69.731441	(61.980976, 77.481907)
	Zhao L (1990)	69.780594	(62.021193, 77.539994)
	Wiedermann CJ (1994)	69.641398	(61.88023, 77.402567)
	Wiedermann CJ (1994)	68.909757	(61.192493, 76.627021)
	Chiarantini E (1996)	69.831981	(62.072623, 77.591339)
	Chiarantini E (1996)	69.572919	(61.817256, 77.328582)
	Chiarantini E (1996)	69.700743	(61.953485, 77.448001)
	Chiarantini E (1996)	69.747927	(61.992072, 77.503782)
	Jaremo P (1996)	70.077942	(62.322536, 77.833349)
	Jaremo P (1996)	70.201283	(62.448488, 77.954078)
	Chiarantini E (1997)	69.532194	(61.776983, 77.287406)
	Heits F (1999)	69.193217	(61.443457, 76.942977)
	Heits F (1999)	69.905601	(62.144745, 77.666456)
	Kapsoritakis A (2001)	70.179739	(62.421022, 77.938456)
	Kapsoritakis A (2001)	70.411289	(62.654614, 78.167965)
	Kapsoritakis A (2001)	69.60064	(61.847336, 77.353944)
	Kapsoritakis A (2001)	70.349046	(62.592578, 78.105515)
	Sturm A (2000)	69.967384	(62.212994, 77.721774)
	Sturm A (2000)	69.970004	(62.211993, 77.728014)
	Kapsoritakis A (2001)	70.231383	(62.471371, 77.991395)
	Kapsoritakis A (2001)	70.487487	(62.730593, 78.244381)
	Kapsoritakis A (2001)	69.697129	(61.940292, 77.453966)
	Kapsoritakis A (2001)	70.374213	(62.616838, 78.131588)
	van Bodegraven AA (2001)	77.636461	(69.877756, 62.119051)
	van Bodegraven AA (2001)	77.901694	(70.140872, 62.38005)
	Dai X (2003)	69.487421	(61.732011, 77.24283)
	Dai X (2003)	70.033735	(62.274993, 77.792477)
	Kapsoritakis A (2003)	70.105252	(62.349601, 77.860904)
	Dong WG (2004)	70.095308	(62.329415, 77.8612)
	Dong WG (2004)	70.294977	(62.531352, 78.058601)
	Irving P (2004)	69.831744	(62.071982, 77.591507)
	Andoh A (2006)	69.552285	(61.800396, 77.304175)
	Andoh A (2006)	69.755129	(61.997488, 77.512769)
	Drzewoski J (2006)	69.343419	(61.591208, 77.095631)
	Drzewoski J (2006)	70.07685	(62.319367, 77.834332)
	Efrat B (2006)	69.71867	(61.961149, 77.476191)

	Payzin B (2006)	69.270722	(61.52016, 77.021285)
	Payzin B (2006)	70.407526	(62.653253, 78.1618)
	Kayahan H (2007)	69.579371	(61.828099, 77.330642)
	Kayahan H (2007)	70.202042	(62.441313, 77.96277)
	Maher MM (2008)	70.086872	(62.329718, 77.844026)
	Maher MM (2008)	69.631365	(61.885265, 77.377465)
	Cakal B (2009)	69.822638	(62.067845, 77.577432)
	Cakal B (2009)	70.291639	(62.535053, 78.048226)
	Cakal B (2009)	69.758213	(62.011324, 77.505102)
	Cakal B (2009)	70.454833	(62.704096, 78.205569)
	Krzystek-Korpacka M (2009)	69.588169	(61.836399, 77.339939)
	Krzystek-Korpacka M (2009)	70.222339	(62.463975, 77.980704)
	Krzystek-Korpacka M (2009)	69.569864	(61.81743, 77.322297)
	Krzystek-Korpacka M (2009)	70.177606	(62.425202, 77.93001)
	Shen J (2009)	69.975767	(62.215087, 77.736446)
	Shen J (2009)	69.782069	(62.026044, 77.538093)
	Shen J (2009)	70.180935	(62.417442, 77.944428)
	Shen J (2009)	69.962075	(62.198468, 77.725682)
	Krzystek-Korpacka M (2010)	69.861195	(62.106778, 77.615613)
	Krzystek-Korpacka M (2010)	70.334436	(62.573704, 78.095169)
	Krzystek-Korpacka M (2010)	69.67614	(61.92606, 77.426221)
	Krzystek-Korpacka M (2010)	70.279198	(62.529697, 78.028699)
	Krzystek-Korpacka M (2010)	69.563272	(61.80989, 77.316654)
	Krzystek-Korpacka M (2010)	70.100869	(62.344274, 77.857464)
	Arhan M (2011)	70.089495	(62.328996, 77.849994)
	Arhan M (2011)	69.847358	(62.08888, 77.605837)
	Dogan Y (2011)	70.214375	(62.452459, 77.976291)
	Mohammadi M (2011)	70.315346	(62.559211, 78.071481)
	Mohammadi M (2011)	70.625657	(62.87096, 78.380355)
	Polinska B (2011)	70.001461	(62.247067, 77.755854)
	Yarur AJ (2011)	69.975392	(62.202625, 77.748159)
	Yesil A (2011)	69.441878	(61.687436, 77.196321)
	Yesil A (2011)	70.229956	(62.469251, 77.990662)
	Yesil A (2011)	69.85481	(62.093052, 77.616569)
	Yesil A (2011)	70.300296	(62.539049, 78.061543)
	Huang Q (2012)	69.560841	(61.803712, 77.317969)
	Huang Q (2012)	70.054897	(62.295892, 77.813902)
	Akdogan RA (2013)	70.163012	(62.403008, 77.923016)
	VoudoEuropeis E (2013)	69.908293	(62.14318, 77.673405)
	VoudoEuropeis E (2013)	69.845585	(62.081998, 77.609173)
	Garg M (2013)	70.233229	(62.474461, 77.991996)
	Garg M (2013)	70.210265	(62.451089, 77.96944)
	Liu W (2013)	70.236179	(62.470661, 78.001698)

	Liu W (2013)	69.772218	(62.007763, 77.536673)
	Liu W (2013)	69.300154	(61.547593, 77.052716)
	Liu W (2013)	70.329102	(62.559894, 78.09831)
	Liu W (2013)	69.825297	(62.055202, 77.595393)
	Liu W (2013)	69.223362	(61.477102, 76.969621)
	Liu W (2013)	70.347558	(62.573184, 78.121931)
	Ozturk Z (2013)	69.698839	(61.938998, 77.458681)
	Ozturk Z (2013)	70.004271	(62.242516, 77.766025)
	Ozturk Z (2013)	69.523117	(61.766734, 77.279501)
	Ozturk Z (2013)	69.908424	(62.146676, 77.670171)
	Schoepfer AM (2013)	69.780778	(62.019825, 77.54173)
	Avdagic N (2014)	69.714497	(61.955669, 77.473325)
	Avdagic N (2014)	70.154819	(62.393943, 77.915695)
	Ciecko-Michalska I (2014)	69.362597	(61.608813, 77.11638)
	Ciecko-Michalska I (2014)	69.614207	(61.857899, 77.370515)
	Dolapcioglu C (2014)	69.018832	(61.419101, 76.618564)
	Dolapcioglu C (2014)	68.50218	(60.930825, 76.073536)
	Huo H (2014)	68.400805	(60.702773, 76.098837)
	Huo H (2014)	70.402543	(62.6369, 78.168187)
	Garg M (2015)	70.13692	(62.379544, 77.894296)
	Garg M (2015)	70.100664	(62.344871, 77.856457)
	Tang J (2015)	69.308479	(61.557265, 77.059693)
	Tang J (2015)	69.758464	(62.000964, 77.515964)
	Cibor D (2017)	69.810331	(62.052629, 77.568033)
	Cibor D (2017)	70.139146	(62.379082, 77.899211)
	Cibor D (2017)	69.707582	(61.949186, 77.465977)
	Cibor D (2017)	70.044361	(62.289091, 77.79963)
	Gawronska B (2017)	69.989601	(62.231878, 77.747323)
	Szczeklik K (2017)	69.05374	(61.403847, 76.703633)
	Ye L (2017)	70.077385	(62.318785, 77.835985)
	Ye L (2017)	70.090009	(62.346198, 77.83382)
	Ye L (2017)	70.113571	(62.357043, 77.8701)
	Ye L (2017)	70.57335	(62.818817, 78.327883)
	Chen G (2018)	69.750696	(61.984577, 77.516814)
	Szczeklik K (2018)	69.726739	(61.974508, 77.47897)
	Szczeklik K (2018)	70.34075	(62.580443, 78.101056)
	Szczeklik K (2018)	70.00673	(62.24696, 77.7665)
	Wang D (2018)	70.095831	(62.334324, 77.857338)
	Wang D (2018)	70.232401	(62.472102, 77.992701)
	Coskun Y (2019)	70.111398	(62.353203, 77.869593)
	Coskun Y (2019)	70.379833	(62.622367, 78.137298)
	Erecan E (2019)	70.289689	(62.531412, 78.047965)
	Jiang Q (2019)	70.090965	(62.274591, 77.907339)

	Kothari HG (2019)	69.927998	(62.168927, 77.68707)
	Bai X (2020)	69.459996	(62.27378, 76.646212)
	Li L (2020)	69.992582	(62.230573, 77.754591)
	Li L (2020)	69.798434	(62.033797, 77.563071)
	Sari C (2020)	69.722545	(61.962717, 77.482374)
	Sari C (2020)	70.218993	(62.455905, 77.982081)
	Chen Z (2021)	70.344142	(62.583701, 78.104583)
	Chen Z (2021)	69.98394	(62.221684, 77.746196)
	Chen Z (2021)	69.599741	(61.84575, 77.353733)
	Chen Z (2021)	70.100913	(62.35179, 77.850036)
	Galijasevic M (2021)	69.82472	(62.065826, 77.583614)
	Galijasevic M (2021)	70.202976	(62.442607, 77.963345)
	Galijasevic M (2021)	69.71015	(61.95416, 77.466139)
	Galijasevic M (2021)	70.366566	(62.608132, 78.124999)
	Zhang G (2021)	70.095695	(62.335378, 77.856012)
	Zhang MH (2021)	70.145079	(62.376036, 77.914122)
	Ammar SR (2022)	70.337631	(62.581297, 78.093966)
	Lu J (2022)	69.893512	(62.100201, 77.686823)
	Basha OM (2023)	70.369267	(62.609392, 78.129142)
	Feng R (2023)	69.389549	(61.635425, 77.143674)
	Geng B (2023)	70.247907	(62.485206, 78.010608)
	Geng B (2023)	69.854651	(62.094665, 77.614637)
	Geng B (2023)	69.473758	(61.718146, 77.229369)
	Hameed NAA (2023)	70.239952	(62.485296, 77.994607)
	Hameed NAA (2023)	69.873005	(59.502127, 80.243883)
	Huang J (2023)	69.916575	(62.147344, 77.685806)
	Huang J (2023)	69.601347	(61.842139, 77.360555)
	Liu Q (2023)	69.80463	(62.046523, 77.562738)
	Sleutjes JAM (2023)	70.237253	(62.459035, 78.015471)
	Xue J (2023)	69.732433	(61.970794, 77.494072)
	Xue J (2023)	69.535949	(61.778463, 77.293434)
	Xue J (2023)	69.991944	(62.226448, 77.75744)
	Ye Y (2023)	70.15419	(62.398353, 77.910026)
	Ye Y (2023)	70.020852	(62.269339, 77.772366)
	Ye Y (2023)	69.729165	(61.977023, 77.481306)
**MPV**			
	Zhao L (1990)	-0.90813	(-1.0834293, -0.73283835)
	Jaremo P (1996)	-0.91238	(-1.0881584, -0.73660468)
	Jaremo P (1996)	-0.92967	(-1.1037467, -0.75558856)
	Kapsoritakis A (2001)	-0.9128	(-1.0888002, -0.73680393)
	Kapsoritakis A (2001)	-0.92212	(-1.097226, -0.74701143)
	Kapsoritakis A (2001)	-0.89921	(-1.0747168, -0.72370642)
	Kapsoritakis A (2001)	-0.91966	(-1.0948233, -0.7444996)

	Kapsoritakis A (2003)	0.914019	(-1.1089059, -0.71913266)
	Irving P (2004)	-0.92221	(-1.0973257, -0.74709818)
	Kayahan H (2007)	-0.90621	(-1.0817004, -0.7307195)
	Kayahan H (2007)	-0.92143	(-1.0965015, -0.74635431)
	Shen J (2009)	-0.90692	(-1.0825403, -0.73130433)
	Shen J (2009)	-0.90583	(-1.080549, -0.73111234)
	Shen J (2009)	-0.9126	(-1.0885666, -0.736633)
	Shen J (2009)	-0.9155	(-1.0895942, -0.74140982)
	Yuksel O (2009)	-0.9187	(-1.0942015, -0.74319672)
	Yuksel O (2009)	-0.92627	(-1.1007929, -0.75174939)
	Arhan M (2011)	-0.92339	(-1.0982201, -0.74856909)
	Arhan M (2011)	-0.91297	(-1.0898555, -0.73609433)
	Dogan Y (2011)	-0.92951	(-1.1031936, -0.75582675)
	Polinska B (2011)	-0.91763	(-1.0932866, -0.7419822)
	Huang Q (2012)	-0.86531	(-1.0347448, -0.69587618)
	Huang Q (2012)	-0.88417	(-1.0577204, -0.71061771)
	Liu S (2012)	-0.89779	(-1.0754529, -0.72013263)
	Liu S (2012)	-0.89998	(-1.0813308, -0.71863596)
	Voudoukis E (2013)	-0.90877	(-1.0859641, -0.73158524)
	Voudoukis E (2013)	-0.91297	(-1.0898147, -0.73612162)
	Liu W (2013)	-0.9192	(-1.0935624, -0.74483337)
	Liu W (2013)	-0.90098	(-1.0761509, -0.72581828)
	Liu W (2013)	-0.85825	(-1.0240357, -0.69246096)
	Liu W (2013)	-0.92733	(-1.1017605, -0.75289299)
	Liu W (2013)	-0.88818	(-1.062304, -0.71405782)
	Liu W (2013)	-0.87238	(-1.0440512, -0.70071118)
	Liu W (2013)	-0.91831	(-1.0933883, -0.74322706)
	Ozturk Z (2013)	-0.91889	(-1.0946371, -0.74314051)
	Ozturk Z (2013)	-0.91449	(-1.0907129, -0.73825954)
	Ozturk Z (2013)	-0.91578	(-1.0916901, -0.73987174)
	Ozturk Z (2013)	-0.9171	(-1.0928307, -0.74135987)
	Tang J (2015)	-0.92106	(-1.0968495, -0.74526483)
	Tang J (2015)	-0.91932	(-1.0947878, -0.74384894)
	Gawronska B (2017)	-0.92318	(-1.0982195, -0.74814852)
	Coskun Y (2019)	-0.91712	(-1.0931261, -0.74111459)
	Coskun Y (2019)	-0.92196	(-1.0972584, -0.74666631)
	Erecan E (2019)	-0.92036	(-1.0951182, -0.74560657)
	Chen Z (2021)	-0.92936	(-1.1034887, -0.75522822)
	Chen Z (2021)	-0.91203	(-1.0883443, -0.73570952)
	Chen Z (2021)	-0.90444	(-1.0791644, -0.72970821)
	Chen Z (2021)	-0.94014	(-1.1073121, -0.77296818)
	Galijasevic M (2021)	-0.91093	(-1.0865188, -0.73533729)
	Galijasevic M (2021)	-0.92178	(-1.0968566, -0.74670267)

	Galijasevic M (2021)	-0.92144	(-1.0964756, -0.74639795)
	Galijasevic M (2021)	-0.93211	(-1.1058286, -0.75838718)
	Zhang G (2021)	-0.91164	(-1.0872412, -0.73604567)
	Basha OM (2023)	-0.89816	(-1.0738931, -0.72242342)
	Feng R (2023)	-0.92232	(-1.0975728, -0.7470572)
**PDW**			
	Shen J (2009)	-0.28256566	(-0.73704063, 0.17190932)
	Shen J (2009)	-0.23902901	(-0.69565829, 0.21760027)
	Shen J (2009)	-0.32515484	(-0.76761082, 0.11730115)
	Shen J (2009)	-0.32119379	(-0.7653786, 0.12299102)
	Voudoukis E (2013)	-0.24719182	(-0.73746843, 0.24308479)
	Voudoukis E (2013)	-0.25722657	(-0.72755162, 0.21309848)
	Ozturk Z (2013)	-0.18239127	(-0.6459956, 0.28121306)
	Ozturk Z (2013)	-0.23261617	(-0.70133822, 0.23610589)
	Ozturk Z (2013)	-0.20610691	(-0.67224656, 0.26003273)
	Ozturk Z (2013)	-0.24497479	(-0.71340286, 0.22345327)
	Avdagic N (2014)	-0.19592982	(-0.66241221, 0.27055256)
	Avdagic N (2014)	-0.22982928	(-0.69789762, 0.23823906)
	Tang J (2015)	-0.12207885	(-0.55388048, 0.30972278)
	Tang J (2015)	-0.18631593	(-0.64587751, 0.27324564)
	Galijasevic M (2021)	-0.16141381	(-0.62086374, 0.29803612)
	Galijasevic M (2021)	-0.2077136	(-0.67550001, 0.26007281)
	Galijasevic M (2021)	-0.11150255	(-0.55865706, 0.33565197)
	Galijasevic M (2021)	-0.17181577	(-0.62710514, 0.28347361)
	Zhang G (2021)	-0.16661281	(-0.62452725, 0.29130163)
	Gerceker E (2023)	-0.14420017	(-0.59248595, 0.30408561)
	Xue J (2023)	-0.13987376	(-0.58388796, 0.30414044)
	Xue J (2023)	-0.17305356	(-0.63867371, 0.29256659)
	Xue J (2023)	-0.28256566	(-0.73704063, 0.17190932)
**PCT**			
	Zhao L (1990)	0.04625	(0.03019712, 0.06230258)
	Kapsoritakis A (2003)	0.048694	(0.03694382, 0.06044342)
	Voudoukis E (2013)	0.046928	(0.03047657, 0.06337861)
	Voudoukis E (2013)	0.045962	(0.02970629, 0.06221811)
	Ozturk Z (2013)	0.043991	(0.02852695, 0.0594557)
	Ozturk Z (2013)	0.046255	(0.03017291, 0.0623379)
	Ozturk Z (2013)	0.044576	(0.02891555, 0.06023632)
	Ozturk Z (2013)	0.046247	(0.03021018, 0.06228287)
	Avdagic N (2014)	0.045445	(0.02955892, 0.06133196)
	Avdagic N (2014)	0.047092	(0.03114897, 0.06303423)
	Tang J (2015)	0.041515	(0.02757438, 0.05545626)
	Tang J (2015)	0.045249	(0.02950402, 0.060994)
	Galijasevic M (2021)	0.046207	(0.03030982, 0.06210417)

	Galijasevic M (2021)	0.047839	(0.0318654, 0.0638119)
	Galijasevic M (2021)	0.043284	(0.02791436, 0.05865284)
	Galijasevic M (2021)	0.048249	(0.03237175, 0.06412528)
	Zhang G (2021)	0.046208	(0.03030747, 0.06210933)
	Huang J (2023)	0.046754	(0.02945056, 0.06405751)
	Huang J (2023)	0.04522	(0.02935277, 0.06108809)

Abbreviations: PLT, platelet count; MPV, mean platelet volume; PDW, platelet distribution width; PCT, plateletcrit; 95% CI: 95% confidence interval

Supplementary Table S7. Egger's Test

	Std_Eff	Coef.	Std. Err.	t	*P*	95% CI
**PLT**	slope	52.08139	3.058144	17.03	<0.001	(46.04157, 58.12122)
	bias	1.635874	.9057901	1.81	0.073	(-0.153058, 3.424806)
**MPV**	slope	-1.304238	0.1162323	-11.22	<0.001	(-1.53737, -1.071105)
	bias	1.761869	0.7609127	2.32	**0.024**	(0.2356718, 3.288067)
**PDW**	slope	0.3233737	0.4180176	0.77	0.448	(-0.5485957, 1.195343)
	bias	-1.757884	1.702361	-1.03	0.314	(-5.308946, 1.793179)
**PCT**	slope	-0.00405	0.007689	-0.53	0.605	(-0.02027, 0.0121759)
	bias	4.58241	1.059564	4.32	**<0.001**	(2.346924, 6.817895)

Abbreviations: PLT, platelet count; MPV, mean platelet volume; PDW, platelet distribution width; PCT, plateletcrit; 95% CI: 95% confidence interval

Supplementary Table S8. The Trim and Fill Method

		**Estimate**	**95% CI**	***P***	**Number of studies**
**MPV**	before	-0.912	(-1.086, -0.739)	**<0.001**	55
	after	-1.139	(-1.307, -0.970)	**<0.001**	68
**PCT**	before	0.046	(0.031, 0.061)	**<0.001**	19
	after	0.015	(0.000, 0.030)	**0.05**	29

Abbreviations: MPV, mean platelet volume; PCT, plateletcrit; 95% CI: 95% confidence interval

Supplementary Figure S1. Forest plot of the difference of platelet parameters between the IBD group and the control group.

A. PLT

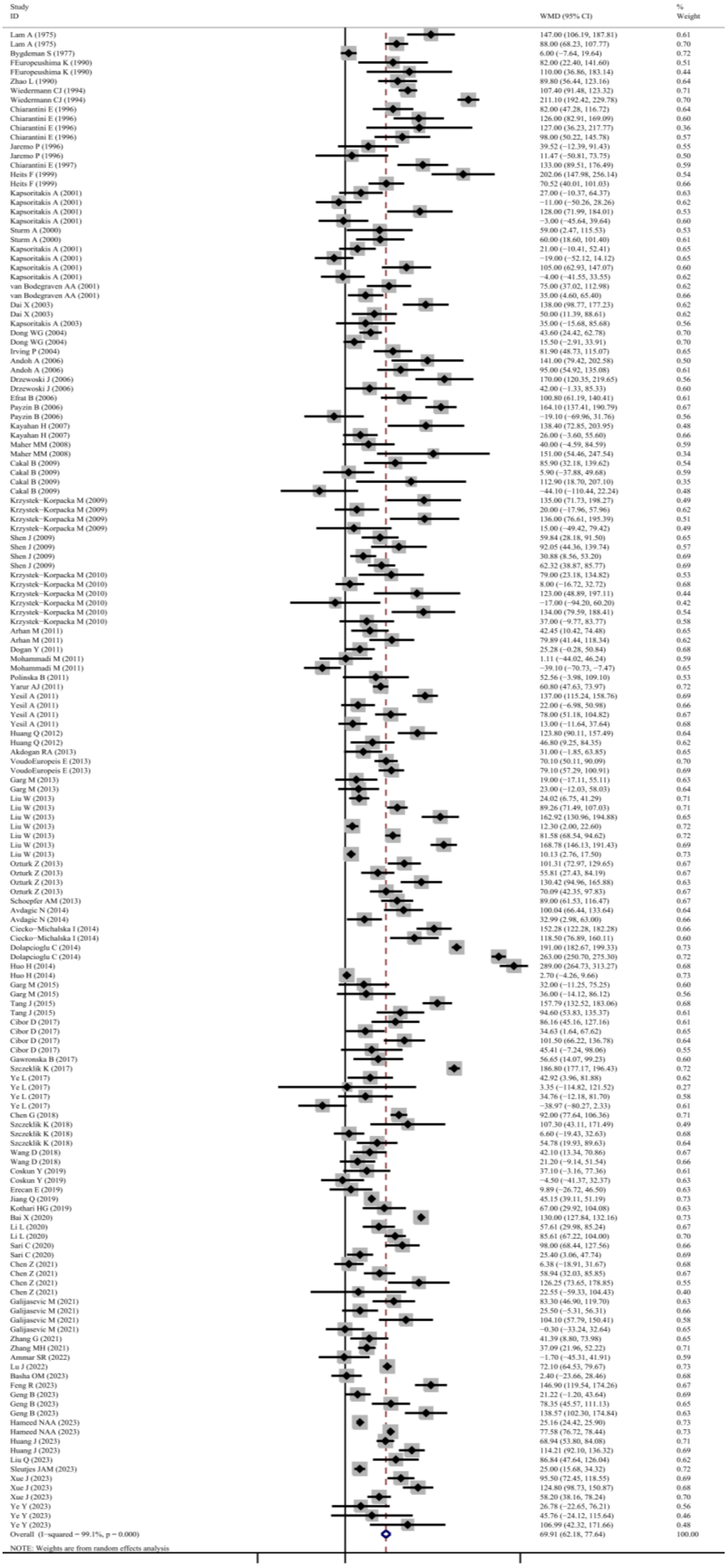

B. MPV

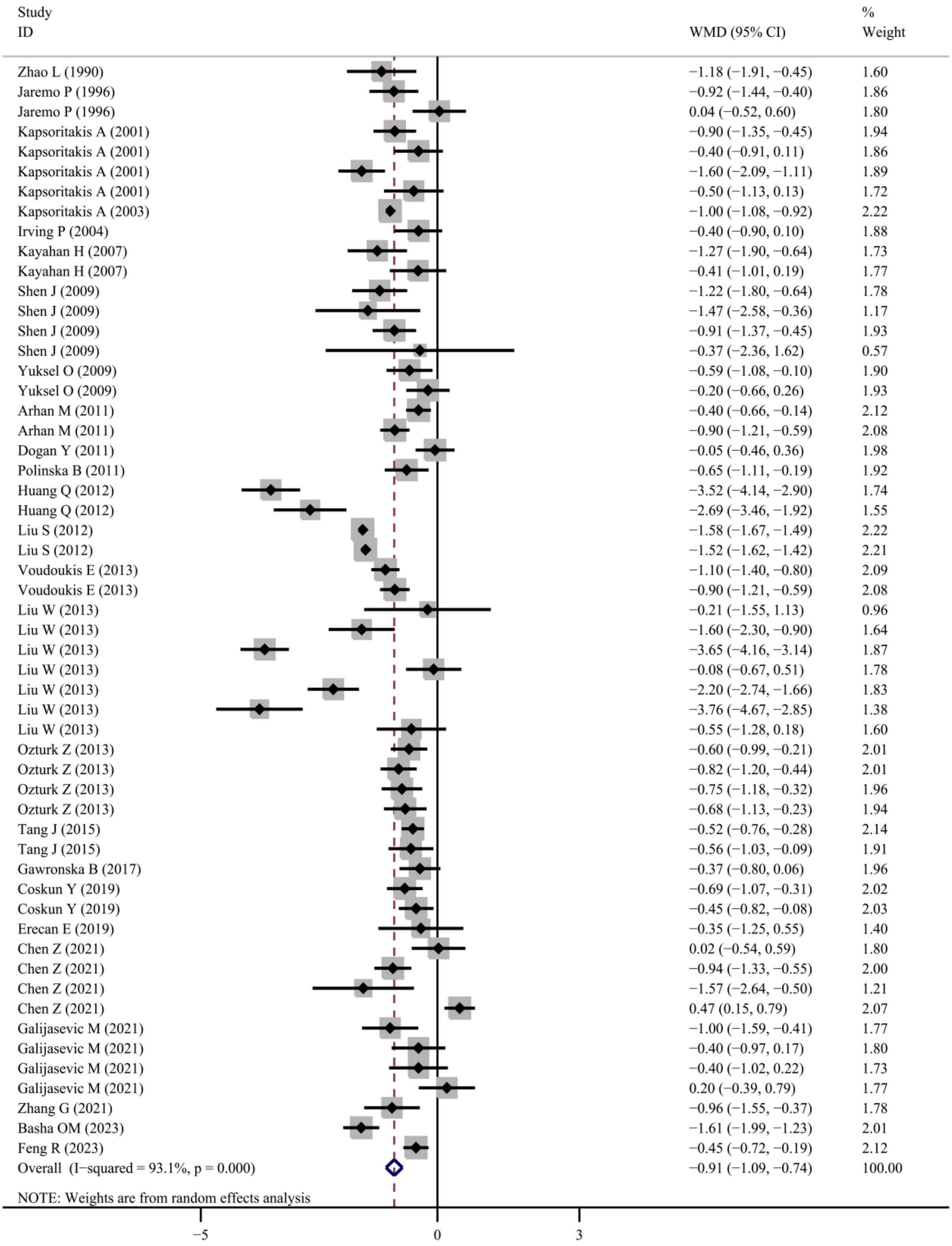
Study
ID
WMD (95% CI)
%
Weight
Zhao L (1990) −1.18 (−1.91, −0.45) 1.60
Jaremo P (1996) −0.92 (−1.44, −0.40) 1.86
Jaremo P (1996) 0.04 (−0.52, 0.60) 1.80
Kapsoritakis A (2001) −0.90 (−1.35, −0.45) 1.94
Kapsoritakis A (2001) −0.40 (−0.91, 0.11) 1.86
Kapsoritakis A (2001) −1.60 (−2.09, −1.11) 1.89
Kapsoritakis A (2001) −0.50 (−1.13, 0.13) 1.72
Kapsoritakis A (2003) −1.00 (−1.08, −0.92) 2.22
Irving P (2004) −0.40 (−0.90, 0.10) 1.88
Kayahan H (2007) −1.27 (−1.90, −0.64) 1.73
Kayahan H (2007) −0.41 (−1.01, 0.19) 1.77
Shen J (2009) −1.22 (−1.80, −0.64) 1.78
Shen J (2009) −1.47 (−2.58, −0.36) 1.17
Shen J (2009) −0.91 (−1.37, −0.45) 1.93
Shen J (2009) −0.37 (−2.36, 1.62) 0.57
Yuksel O (2009) −0.59 (−1.08, −0.10) 1.90
Yuksel O (2009) −0.20 (−0.66, 0.26) 1.93
Arhan M (2011) −0.40 (−0.66, −0.14) 2.12
Arhan M (2011) −0.90 (−1.21, −0.59) 2.08
Dogan Y (2011) −0.05 (−0.46, 0.36) 1.98
Polinska B (2011) −0.65 (−1.11, −0.19) 1.92
Huang Q (2012) −3.52 (−4.14, −2.90) 1.74
Huang Q (2012) −2.69 (−3.46, −1.92) 1.55
Liu S (2012) −1.58 (−1.67, −1.49) 2.22
Liu S (2012) −1.52 (−1.62, −1.42) 2.21
Voudoukis E (2013) −1.10 (−1.40, −0.80) 2.09
Voudoukis E (2013) −0.90 (−1.21, −0.59) 2.08
Liu W (2013) −0.21 (−1.55, 1.13) 0.96
Liu W (2013) −1.60 (−2.30, −0.90) 1.64
Liu W (2013) −3.65 (−4.16, −3.14) 1.87
Liu W (2013) −0.08 (−0.67, 0.51) 1.78
Liu W (2013) −2.20 (−2.74, −1.66) 1.83
Liu W (2013) −3.76 (−4.67, −2.85) 1.38
Liu W (2013) −0.55 (−1.28, 0.18) 1.60
Ozturk Z (2013) −0.60 (−0.99, −0.21) 2.01
Ozturk Z (2013) −0.82 (−1.20, −0.44) 2.01
Ozturk Z (2013) −0.75 (−1.18, −0.32) 1.96
Ozturk Z (2013) −0.68 (−1.13, −0.23) 1.94
Tang J (2015) −0.52 (−0.76, −0.28) 2.14
Tang J (2015) −0.56 (−1.03, −0.09) 1.91
Gawronska B (2017) −0.37 (−0.80, 0.06) 1.96
Coskun Y (2019) −0.69 (−1.07, −0.31) 2.02
Coskun Y (2019) −0.45 (−0.82, −0.08) 2.03
Erecan E (2019) −0.35 (−1.25, 0.55) 1.40
Chen Z (2021) 0.02 (−0.54, 0.59) 1.80
Chen Z (2021) −0.94 (−1.33, −0.55) 2.00
Chen Z (2021) −1.57 (−2.64, −0.50) 1.21
Chen Z (2021) 0.47 (0.15, 0.79) 2.07
Galijasevic M (2021) −1.00 (−1.59, −0.41) 1.77
Galijasevic M (2021) −0.40 (−0.97, 0.17) 1.80
Galijasevic M (2021) −0.40 (−1.02, 0.22) 1.73
Galijasevic M (2021) 0.20 (−0.39, 0.79) 1.77
Zhang G (2021) −0.96 (−1.55, −0.37) 1.78
Basha OM (2023) −1.61 (−1.99, −1.23) 2.01
Feng R (2023) −0.45 (−0.72, −0.19) 2.12
Overall (I−squared = 93.1%, p = 0.000) −0.91 (−1.09, −0.74) 100.00
NOTE: Weights are from random effects analysis
−5
0
3

C. PDW

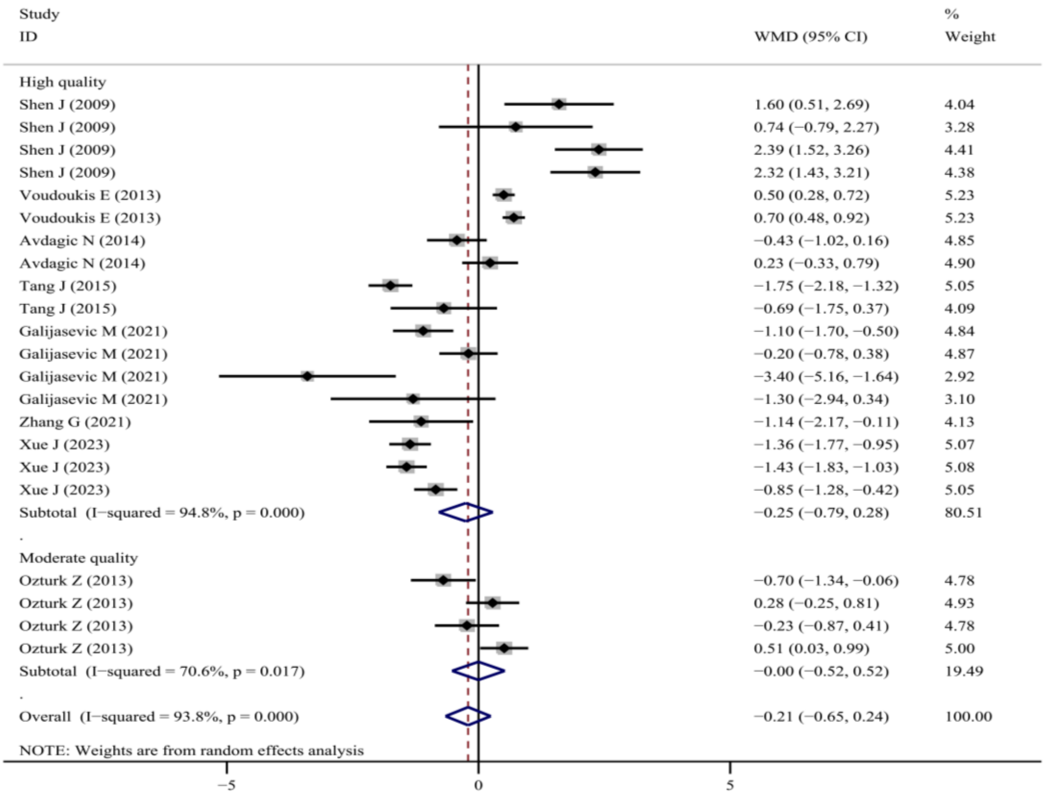

D. PCT

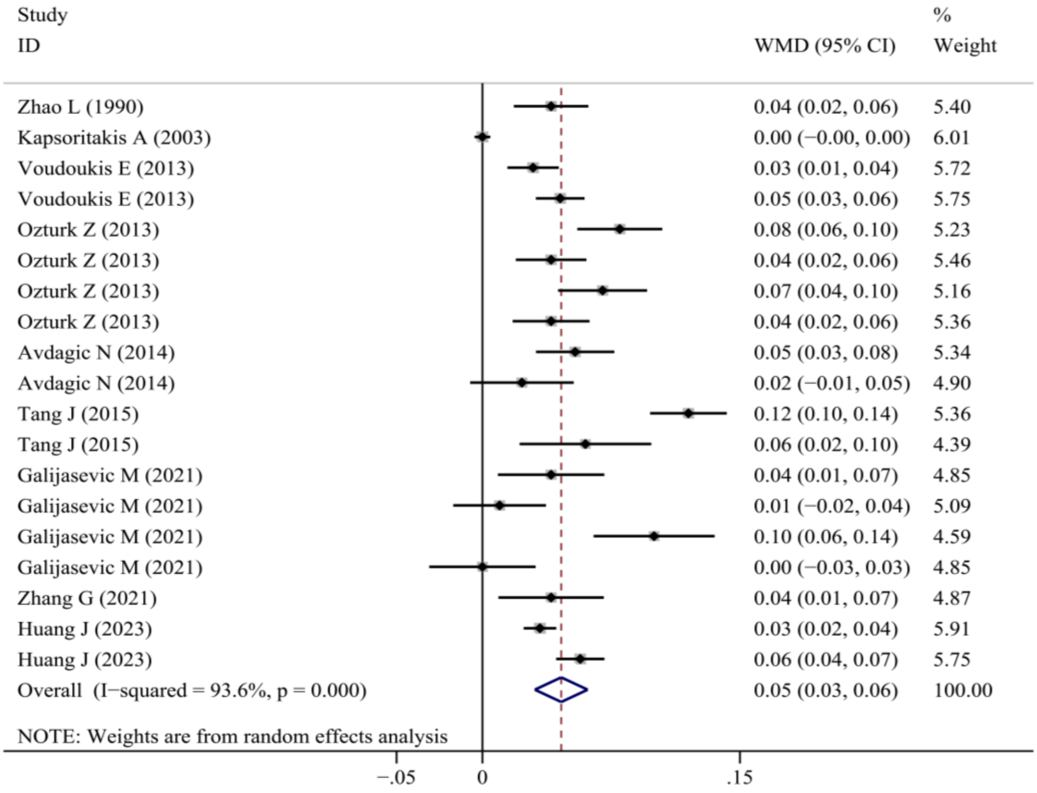

Abbreviations: PLT, platelet count; MPV, mean platelet volume; PDW, platelet distribution width; PCT, plateletcrit; 95% CI: 95% confidence interval

Supplementary Figure S2. Funnel plot of the difference of platelet parameters between the IBD group and the control group.

A. PLT

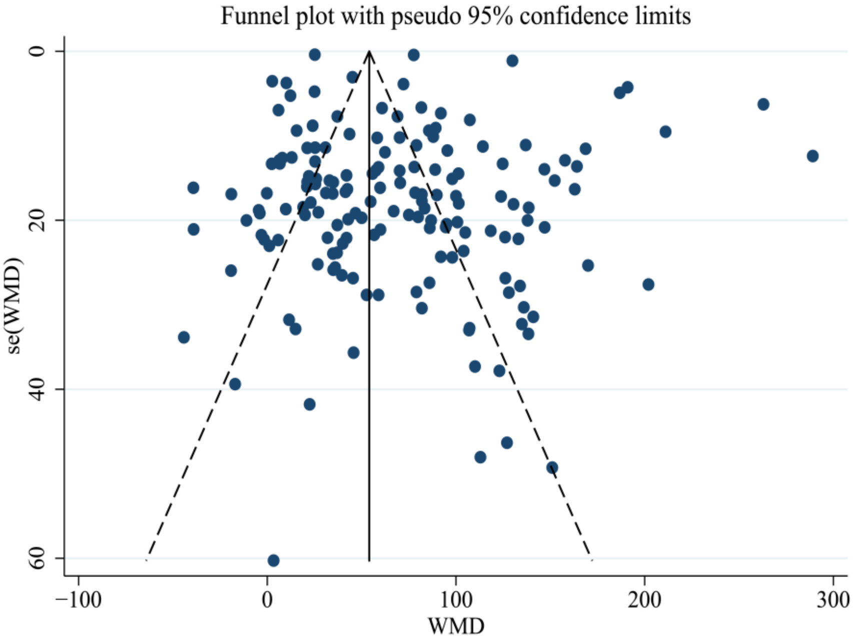

B. MPV

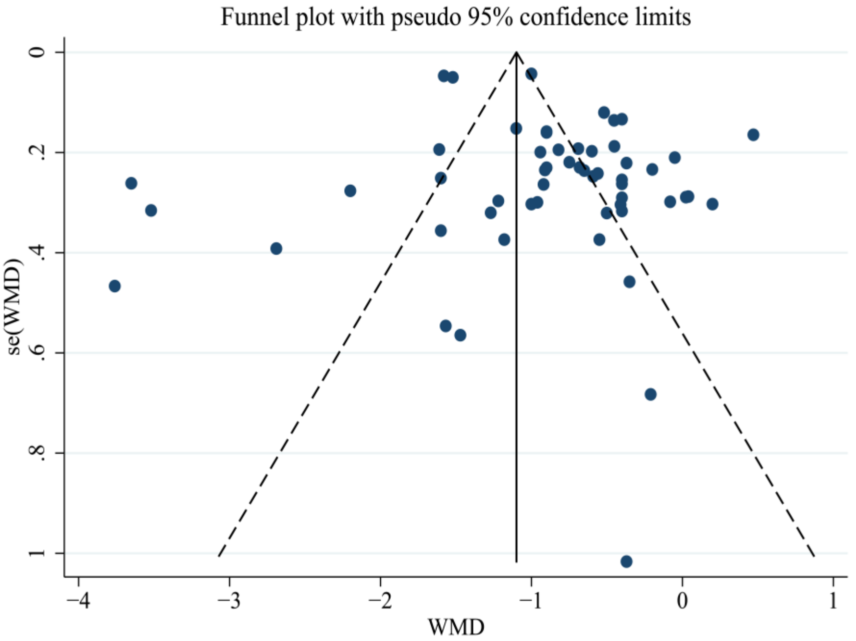

C. PDW

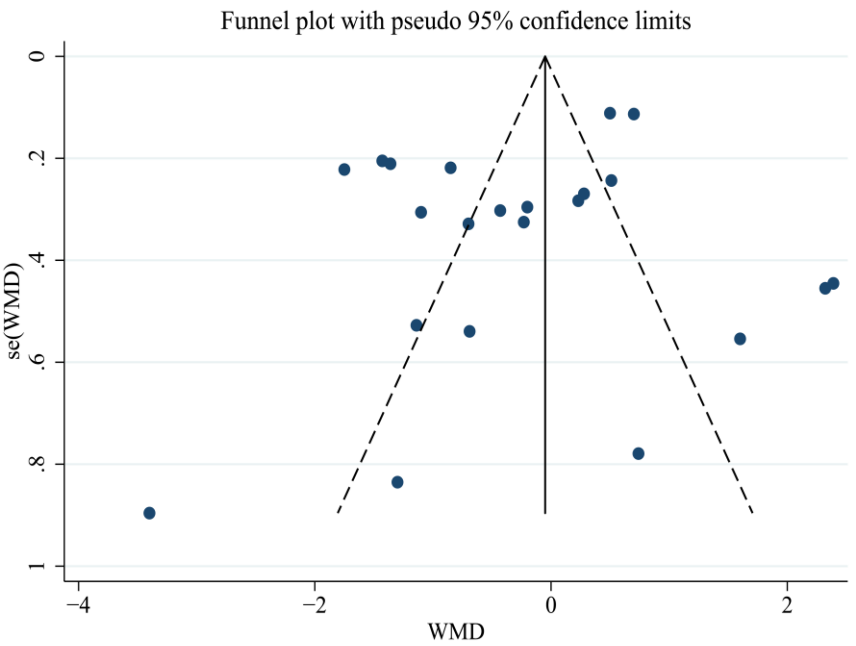

D. PCT

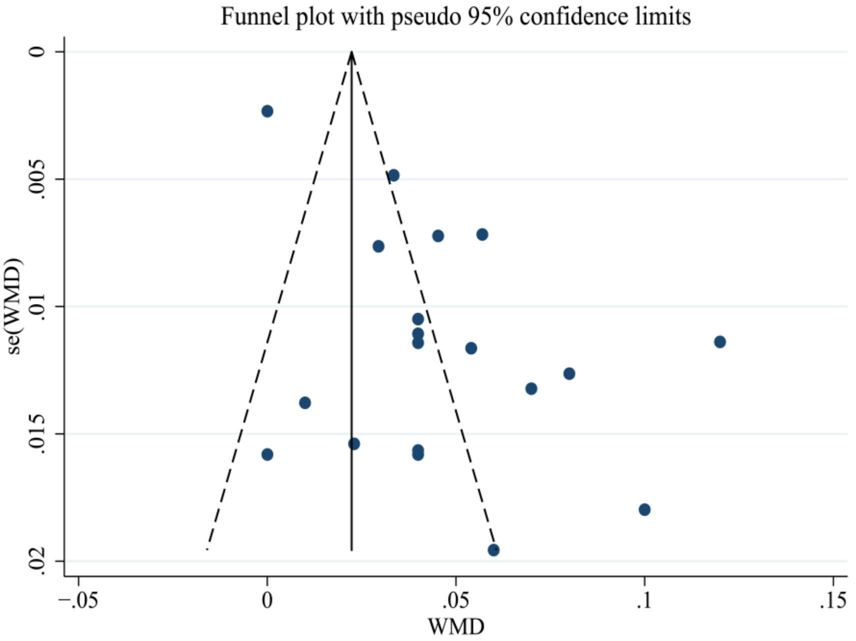

Abbreviations: PLT, platelet count; MPV, mean platelet volume; PDW, platelet distribution width; PCT, plateletcrit

Supplementary Figure S3. Egger's publication bias plot of the difference of platelet parameters between the IBD group and the control group.

A. PLT

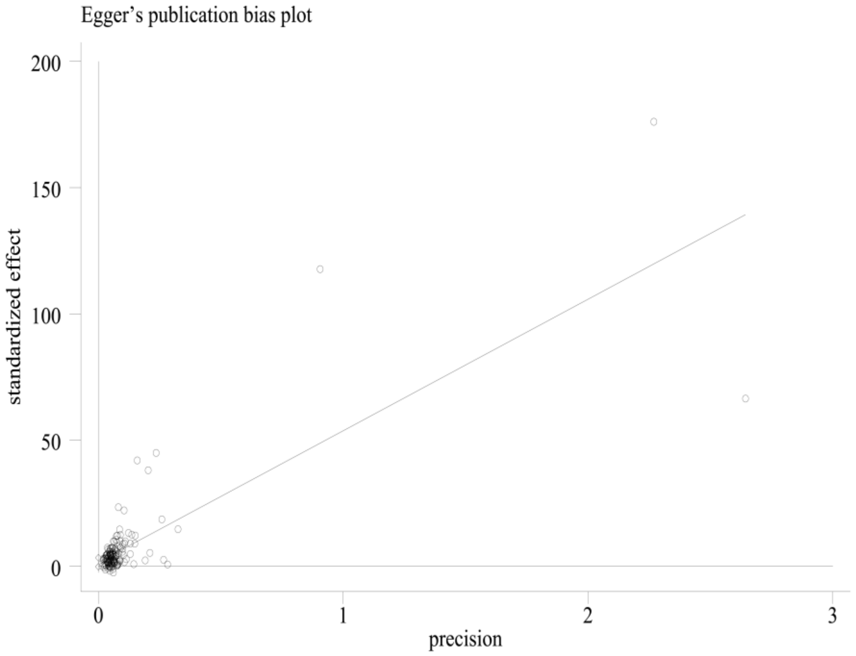

B. MPV

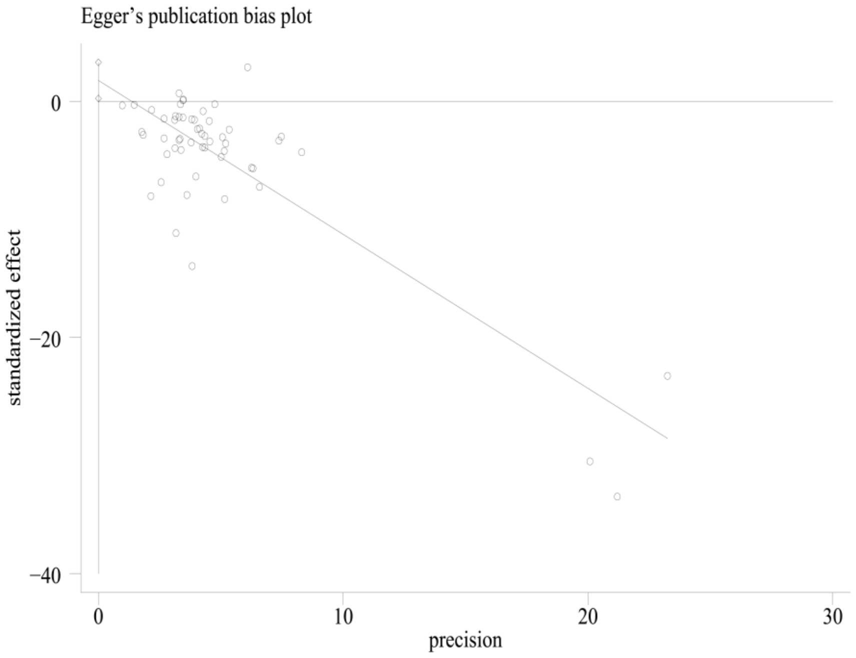

C. PDW

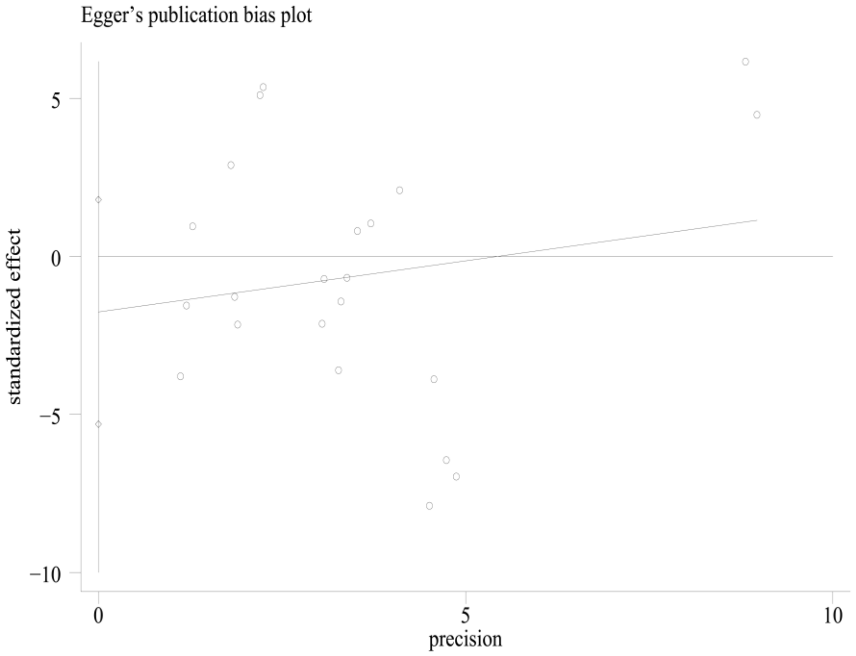

D. PCT

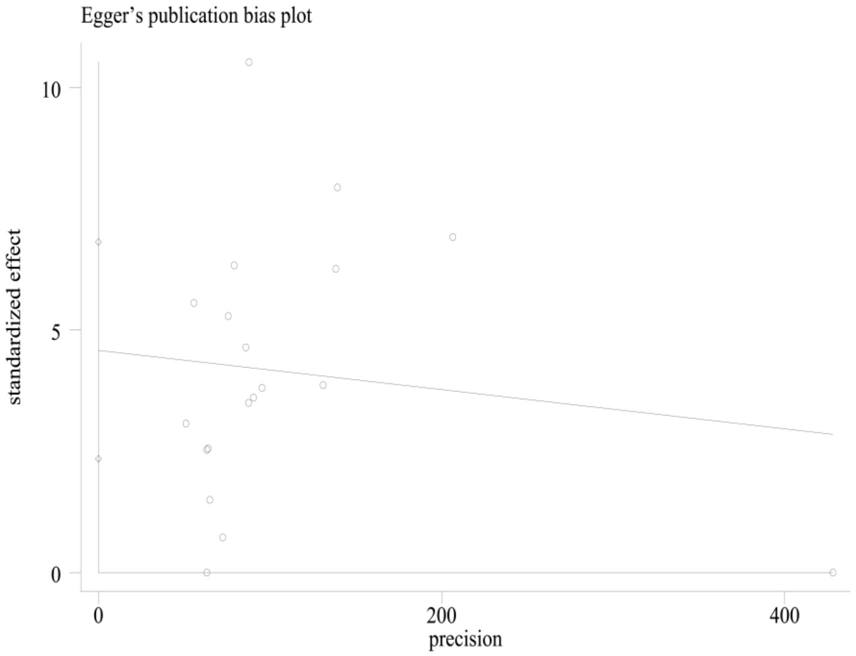

Abbreviations: PLT, platelet count; MPV, mean platelet volume; PDW, platelet distribution width; PCT, plateletcrit

Supplementary Figure S4. Filled funnel plot of the difference of MPV and PCT between the IBD group and the control group.

A. MPV

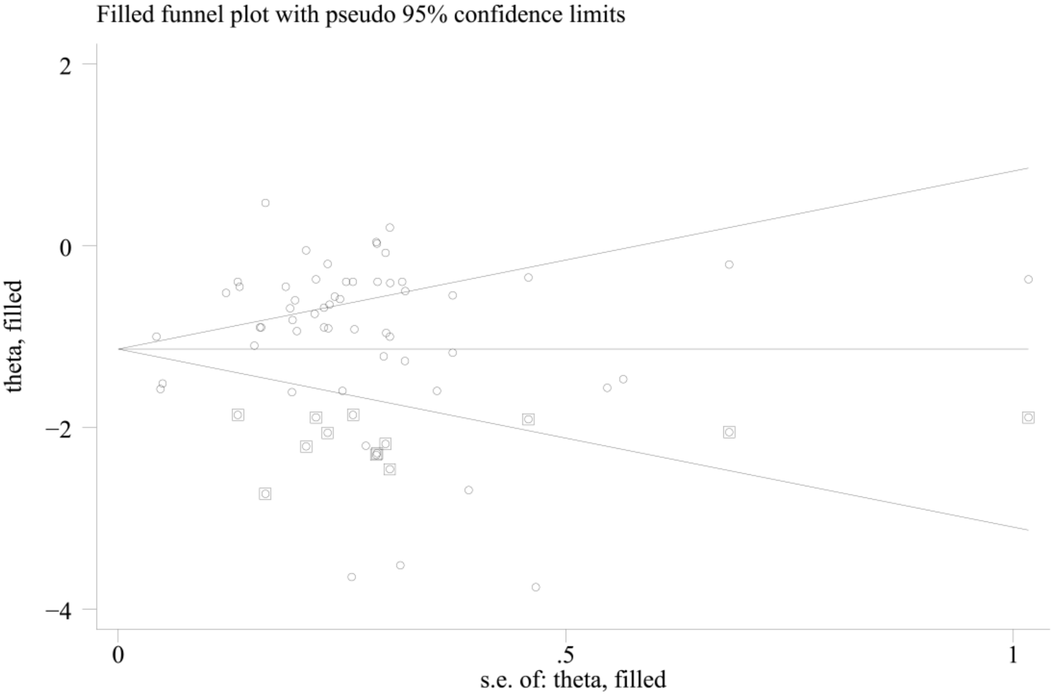

B. PCT

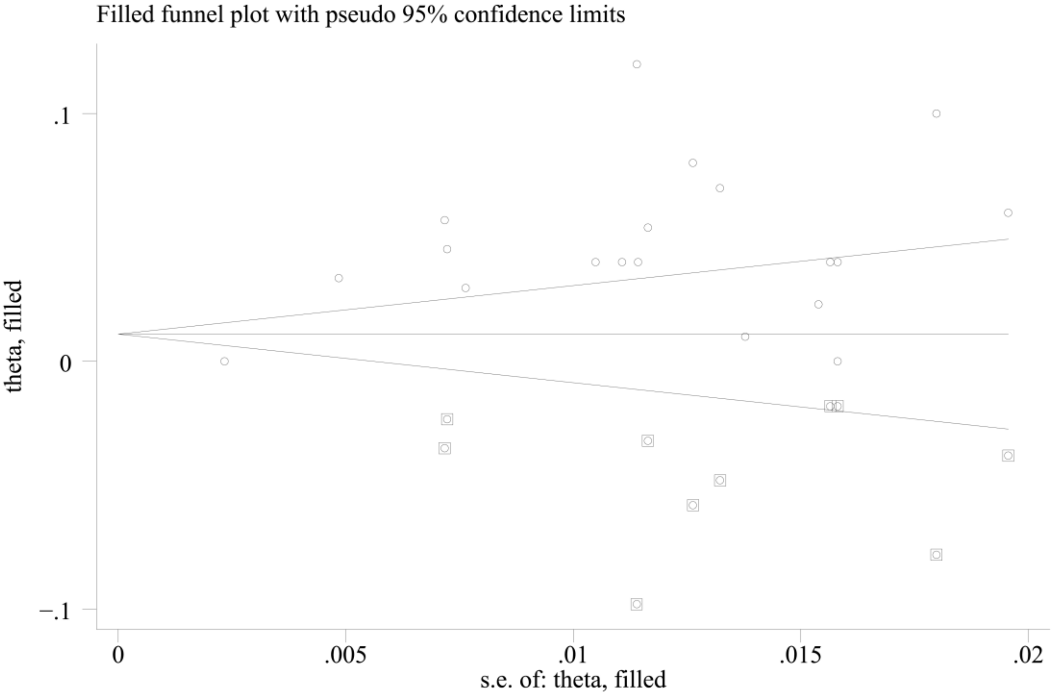

Abbreviations: MPV, mean platelet volume; PCT, plateletcrit
